# Exploring the Impact of Food on the Gut Ecosystem Based on the Combination of Machine Learning and Network Visualization

**DOI:** 10.3390/nu9121307

**Published:** 2017-12-01

**Authors:** Hideaki Shima, Shizuka Masuda, Yasuhiro Date, Amiu Shino, Yuuri Tsuboi, Mizuho Kajikawa, Yoshihiro Inoue, Taisei Kanamoto, Jun Kikuchi

**Affiliations:** 1 The Laboratory of Microbiology, Showa Pharmaceutical University, Machida, Tokyo 194-8543, Japan; shima@ac.shoyaku.ac.jp (H.S.); a12108@ug.shoyaku.ac.jp (S.M.); kajikawa@ac.shoyaku.ac.jp (M.K.); kanamoto@ac.shoyaku.ac.jp (T.K.); 2 RIKEN Center for Sustainable Resource Science, 1-7-22 Suehiro-cho, Tsurumi-ku, Yokohama, Kanagawa 230-0045, Japan; yasuhiro.date@riken.jp (Y.D.); amiu.shino@riken.jp (A.S.); yuuri.tsuboi@riken.jp (Y.T.); 3 Graduate School of Medical Life Science, Yokohama City University, 1-7-29 Suehiro-cho, Tsurumi-ku, Yokohama, Kanagawa 230-0045, Japan; 4 Laboratory of Pharmaceutical Sciences and Education, Showa Pharmaceutical University, Machida, Tokyo 194-8543, Japan; inoue@ac.shoyaku.ac.jp; 5 Graduate School of Bioagricultural Sciences, Nagoya University, 1 Furo-cho, Chikusa-ku, Nagoya, Aichi 464-0810, Japan

**Keywords:** gut ecosystem, food intake, metabolic response, machine learning, network analysis

## Abstract

Prebiotics and probiotics strongly impact the gut ecosystem by changing the composition and/or metabolism of the microbiota to improve the health of the host. However, the composition of the microbiota constantly changes due to the intake of daily diet. This shift in the microbiota composition has a considerable impact; however, non-pre/probiotic foods that have a low impact are ignored because of the lack of a highly sensitive evaluation method. We performed comprehensive acquisition of data using existing measurements (nuclear magnetic resonance, next-generation DNA sequencing, and inductively coupled plasma-optical emission spectroscopy) and analyses based on a combination of machine learning and network visualization, which extracted important factors by the Random Forest approach, and applied these factors to a network module. We used two pteridophytes, *Pteridium aquilinum* and *Matteuccia struthiopteris*, for the representative daily diet. This novel analytical method could detect the impact of a small but significant shift associated with *Matteuccia struthiopteris* but not *Pteridium aquilinum* intake, using the functional network module. In this study, we proposed a novel method that is useful to explore a new valuable food to improve the health of the host as pre/probiotics.

## 1. Introduction

The gastrointestinal tract has a wide variety of functions, mainly in food digestion and nutrient absorption as well as in the development of systemic immunity, regulation of behavior, and targeting of drug delivery [1,2,3]. Gastrointestinal functions are directly and/or indirectly augmented by the gut microbiota [4,5,6,7]. However, dysbiosis can occur, which involves the disruption of proper microbial function; a typical case of this results from consuming an unbalanced diet, resulting in undesirable conditions in the host, such as inflammatory bowel disease and obesity [8,9,10]. Therefore, it is important to obtain a detailed understanding of the interaction between the gastrointestinal tract and intestinal microbiota.

The intestinal microbiota consists of many bacterial species, whose composition and metabolic function responds to the daily diet; probiotics and prebiotics positively impact the health of an individual [11,12]. Additionally, specific bacteria play a role in digesting specific molecules and providing metabolites to the host in cooperation with other bacteria, which are regulated by diet [13,14,15]. Any component of the daily diet and additional food have the potential of developing a pre/probiotic effect, thus emphasizing the need of a precise evaluation method to determine the impact of food on the intestinal microbiota; this information can be utilized to regulate the intestinal microbiota by controlling the daily diet (pre/probiotics), meal frequency, and time of food intake [16,17,18].

Many researchers have attempted to clarify effect of microbiota to host responses using mice, because the results obtained from such studies could be applied to humans [19,20]. Although many researchers have attempted to determine the factors that impact the intestinal microbiota, including antibiotics, pre/probiotics, and food intake, the data obtained from the microbiota analysis are affected by daily diet, stress, environment, and individual characteristics, including gene-based factors [11,15,21,22,23]. To overcome these problems, intestinal microbiota and host responses were measured by multiple methods, including next-generation DNA sequencing (NGS), nuclear magnetic resonance (NMR), mass spectrometry (MS), and biochemical analyses [10,24,25,26,27,28]. However, the data subjected to a multivariate analysis, including principal component analysis (PCA), correlation analysis, factor analysis, clustering analysis, and network analysis [26,27,29,30], remain potential options for further improvement of data mining.

**Figure 1:**
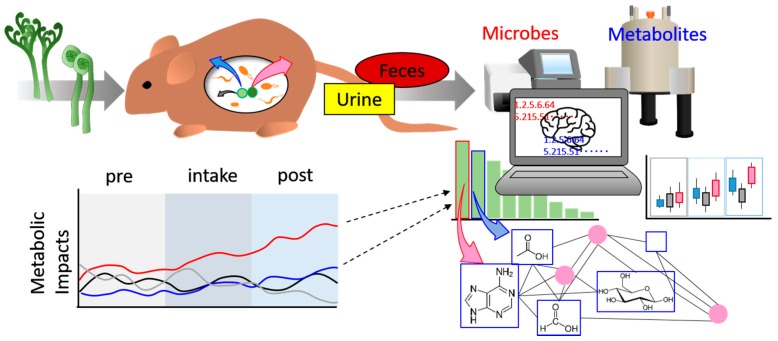
Schematic illustration of the novel method for exploring the impact of food, as proposed in this study. Mice were fed additional food and samples were collected (urine, feces). The samples were measured by multiple methods (e.g., NGS for the microbiome, NMR for the metabolome). These multiple data were analyzed by machine learning and important factors of response to food intake were determined. Furthermore, we calculated the network of normalized multiple data. Finally, combined functional network and important factors revealed masked impacts of food intake.

In this research, we selected *Pteridium aquilinum* and *Matteuccia struthiopteris* as the representative components of the daily diet. *P. aquilinum* is consumed in Japan, Korea, and China, while *M. struthiopteris* is widely consumed in the Northern hemisphere; both plants are pteridophytes, which may have varied nutrient compositions from those of land plants and seaweed. This suggests that pteridophytes have a comparatively simple nutrient profile compared with higher plants, thus enabling easy evaluation [31]. In addition, *P. aquilinum* and *M. struthiopteris* may be beneficial prebiotics in daily diet because of their easy availability (Figure S1) [31]. We chose Random Forest, which is a machine learning tool, superior to classification by decision tree, to identify important factors associated with the impact of food, such as age and geography, on intestinal microbiota composition and applied these important factors to the network community of heterogeneous measurement data (Figure 1) [32,33,34]. This combination of machine learning and network visualization methods works well in identifying significant factors that are difficult to detect by other analytical approaches. A flow chart of the analysis protocol is presented in supplementary Figure S2, which indicates the novel part of this study.

## 2. Materials and Methods

### 2.1. Animals

Male C57BL/6NCrl mice (3 weeks old) were obtained from Oriental Yeast (Tokyo, Japan) and habituated to the conditions in the animal facility before the start of the experiment (at 8 weeks old). All animal experiments were carried out in accordance with the Guidelines for the Laboratory Animal Facility of Showa Pharmaceutical University (ethic code number: P-2015-10).

### 2.2. Food Preparation

*P. aquilinum* and *M. struthiopteris*, used in this study, were developed in Yamagata Prefecture. *P. aquilinum* was boiled in 3% NaHCO_3_ water and then soaked for at least 3 h in the same solution. Soaked *P. aquilinum* was rinsed thrice in sterile water and soaked in new sterile water for over 3 h. *M. struthiopteris* was boiled in 2% NaCl solution for 1 min and then soaked in cool water. These materials were then freeze-dried and crushed.

### 2.3. Animal Experiment and Sample Collection

We fixed the food-intake period (2 weeks) and the pre/post-food-intake periods. Cellulose (Wako), dried *P. aquilinum*, and *M. struthiopteris* were re-suspended (50 mg/mL) in 0.9% NaCl solution. Next, 400 µL of these solutions were orally administered to mice three times every other day in a week. Biological samples (feces, urine) were collected on the same day of food intake. A total of 216 fecal and 183 urinary samples were collected from mice (*n* = 4 for each group) throughout the experimental period for metabolic and microbial analyses. These samples were stored at −80 °C until sample preparation for analysis.

### 2.4. NMR Measurements

For the characterization of the components of *P. aquilinum* and *M. struthiopteris*, freeze-dried powder was suspended in 1 mL of KPi/D_2_O solvent with 1 mmol/L sodium 2, 2-dimethyl-2-silapentane-5-sulfonate (DSS) and heated at 65 °C for 15 min with shaking (1400 rpm). After centrifugation, the supernatants of water- and methanol-soluble components were collected for NMR measurements. For the extraction of macromolecular components, 100 mg of the powder was rinsed with hexafluoroacetone (HFA) and ultrapure water, with a subsequent milling step, in accordance with a slightly modified version of the protocol in a previous study [35]. The milled samples were dissolved in 600 µL of DMSO-d_6_/pyridine-d_5_ (4:1) and the supernatant was used for NMR measurements. The collected urine and feces were prepared for NMR measurements, in accordance with the protocol used in a previous study [36]. All samples were measured by AVANCE II 700 MHz Bruker BioSpin (Bruker, Rheinstetten, Germany), metabolite annotations were performed using ^1^H-^13^C heteronuclear single quantum coherence (HSQC), HSQC-total correlation spectroscopy (TOCSY), double-quantum single-quantum (DQ-SQ), and two-dimensional (2D) ^1^H-^1^H *J*-resolved NMR spectroscopy. In the HSQC NMR measurements, the Bruker standard pulse program “hsqcetgpsisp2.2” was used with the following parameters: number of data points, 1024 (F2) and 256 (F1); number of scans, 128; spectral widths, 9803.922 (F2) and 24,649.248 (F1) Hz; and D1, 1 s. In the HSQC-TOCSY NMR measurements, the Bruker standard pulse program “hsqcdietgpsisp.2” was used with the following parameters: number of data points, 2048 (F2) and 256 (F1); number of scans, 128; and spectral widths, 9803.922 (F2) and 28,170.570 (F1) Hz. In the DQ-SQ NMR measurements, the Bruker standard pulse program “dqseagp90” was used with the following parameters: number of data points, 16,384 (F2) and 512 (F1); number of scans, 64; spectral widths, 9803.0922 (F2) and 14,003.065 (F1) Hz; and D1, 1 s. In the 2D *J*-resolved NMR measurements, the Bruker standard pulse program “jresgpprqf” was used with the following parameters: number of data points, 16,384 (F2) and 32 (F1); number of scans, 8; spectral widths, 12,500 (F2) and 50 (F1) Hz; and D1, 2 s. In addition, metabolic profiling for feces and urine was also performed using 2D *J*-resolved NMR with skyline projection [37]. The detected peaks in NMR spectra were annotated using SpinAssign (http://dmar.riken.jp/spinassign/) and SpinCouple (http://dmar.riken.jp/spincpl/) programs, as well as the Human Metabolome Database (http://www.hmdb.ca/) [38,39,40].

### 2.5. Inductively Coupled Plasma-Optical Emission Spectroscopy (ICP-OES) Measurement

A sufficient amount of each of 138 urinary samples was diluted approximately 100 times and measured by ICP-OES (SPECTRO BLUE FMX26 EOP; SPECTRO, Tokyo, Japan). As the operating parameters, main argon pressure, plasma power, plasma gas flow, auxiliary gas flow, nebulizer gas flow, and pump speed were set to 6.5 bar, 1400 W, 12 L/min, 1 L/min, 1 L/min, and 30 rpm, respectively.

### 2.6. Fecal Microbiome Analysis

Fecal microbes were disrupted using 5 mm stainless beads and a medicine spoonful of 0.3 mm zirconia beads in 200 µL of SDS-TE buffer (10 mmol/L Tris-HCl, 1 mmol/L EDTA, 2% SDS) using a vortex mixer for a total of 60 s. Disrupted feces were subjected to three freeze–thaw cycles and then centrifuged (10,000× *g*, 4 °C, 5 min), after which 100 µL of the supernatant was transferred to a new tube. DNA was extracted by phenol:chloroform:isoamyl alcohol (25:24:1) from the supernatant. Extracted DNA was dissolved in TE buffer and stored at −80 °C until microbiome analysis. The region encoding 16S rRNA was amplified using PCR with a TaKaRa Ex Taq HS kit (TaKaRa Bio, Shiga, Japan) and a primer set (Table S1) [41]. DNA was amplified by the following program: preheating at 94 °C for 4 min; 25 cycles of denaturation at 94 °C for 30 s, annealing at 55 °C for 30 s, and extension at 72 °C for 2 min; and a final terminal extension at 72 °C for 10 min. The 16S rRNA gene amplicon was purified using AMPure XP (Beckman Coulter, Brea, CA, USA) with the manufacturer’s protocol. The purified double-stranded DNA concentration was determined by Picogreen (Thermo Fisher Scientific, Waltham, MA, USA) and sequencing of prepared library was performed using a MiSeq sequencer (Illumina Inc., San Diego, CA, USA) following the manufacturer’s instructions. Obtained DNA sequence data were analyzed by QIIME (http://qiime.org/) and detected bacteria are listed in Table S2 [42]. These bacterial data were handled as percentages in the following analysis. The sequences that passed the QIIME of 16S rRNA OTU had a mean of 5619.26, and SD of 1653.89.

### 2.7. Data Analysis

Peaks of one-dimensional skyline projection (^1^H-NMR) data were picked up by rNMR on the “R” platform [43,44]. The peak-picking data and other microbial and elemental data were normalized by unit variance. PCA, Random Forest, and network analyses were performed using the packages “muma,” “randomForest,” and “igraph” on the R platform, respectively [45,46,47]. These packages were used with their default settings. Calculated network data were depicted by Cytoscape (http://www.cytoscape.org/) [48]. The top 30 important factors were extracted from the mean decrease in accuracy of the Random Forest analysis. These factors were applied to calculate the community. The important factors of the included community were statistically analyzed. Statistical significance was determined using the Holm method, in which the false discovery rate was intermediate between Benjamini–Hochberg method and Bonferroni method [49,50].

### 2.8. Data Deposition

DNA sequences of the study have been deposited in DDBJ (http://www.ddbj.nig.ac.jp/). The accession number was DRA006349.

## 3. Results and Discussion

### 3.1. Sample Measurement and Multivariate Analysis

**Figure 2:**
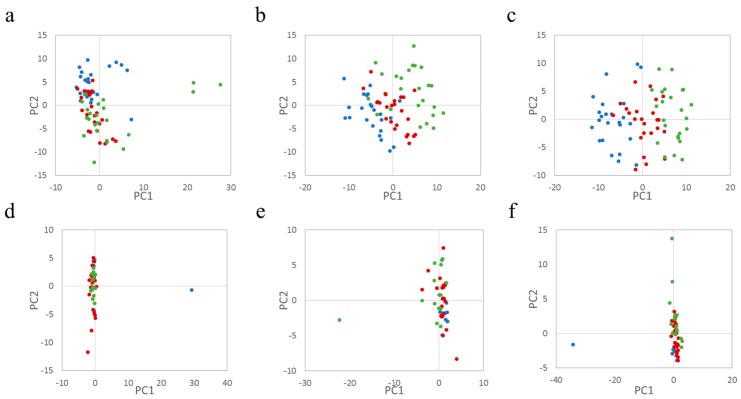
Principal component analysis of fecal metabolites and the microbiome. Red, blue, and green spheres represent single data points from pretreatment, during treatment, and posttreatment, respectively. Upper panels indicate the results of fecal NMR measurement analyzed by PCA. The data for the experimentally treated mouse groups (**a**–**c**) were from the administration of cellulose, *Pteridium aquilinum*, and *Matteuccia struthiopteris*, respectively; (**d**–**f**) show PCA score plots of the MiSeq data for each group; (**d**–**f**) show the results for the microbiota of mice treated with cellulose, *P. aquilinum*, and *M. struthiopteris*, respectively. The data analysis was performed using the package “muma” in R [45].

NMR measurements of the components of *P. aquilinum* and *M. struthiopteris* revealed that the plants contained some sugar groups and organic acids, chlorogenic acid, shikimate, and quinate (Figure S3a,b), the metabolites of which are derived from the shikimate pathway [51]. In particular, chlorogenic acid was reported to reduce blood pressure, indicating that the plants have the possibility of improving health [52]. Additionally, the plants have almost the same high-molecular-weight components, except for olefin and starch (Figure S4a,b). Next, the contents of urine and feces from throughout the experimental period were annotated and used to create a PCA score plot, which is widely used to obtain an overview of multivariate data for easy visualization (Table S3, Figures S5 and S6, Figure 2a–c). Although the PCA score of feces from cellulose-treated mice moved slightly in the PC2 negative direction from the pre to post period (Figure 2a), the PCA scores of *P. aquilinum* and *M. struthiopteris* transitioned from the PC1 negative to the positive side (Figure 2b,c). This suggested that *P. aquilinum* and *M. struthiopteris* intake had a greater impact than cellulose intake, which may have arisen from the plants’ sugar groups and organic acids. However, we were unable to determine the exact causal factor from the PCA score loading, as there was substantial noise. In addition, we measured intestinal microbiota by MiSeq and analyzed the data using PCA. The results of the microbiome data from all groups were not clearly separated among the groups (Figure 2d–f). Furthermore, we estimated the Firmicutes/Bacteroidetes ratio, which are major phyla in the gut and indicators of host life-style [12], to be almost the same (mean = 1.13, SD = 0.42, for the whole experimental period). These results suggest that additional food intake of six times every other day for two weeks could not make a major difference to gut microbiota composition. The urinary profiles based on NMR and elements by ICP-OES indicated a complex dispersion on the PCA score plot (Figure S7a–f). These results appear to demonstrate that PCA was unsuitable for exploring the impact of food in this case.

### 3.2. Selection of Important Variables by Determining the Food Impact

**Figure 3:**
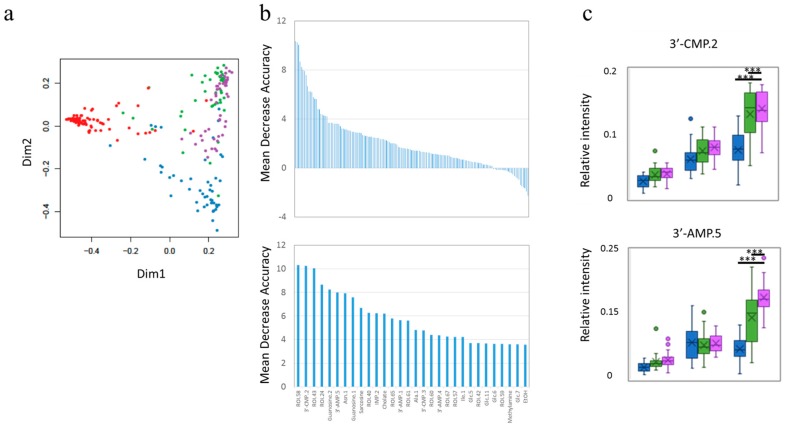
The results of Random Forest for fecal metabolites. Multi-dimensional score (MDS) plots (**a**) and important factors separating the groups (**b**) upper panel indicates all important factors and lower panel indicates the top 30 most important factors with annotation. In (**a**), red, blue, green, and purple circles represent single data points from the pre-treatment period for all groups, and the cellulose-, *Pteridium aquilinum*-, and *Matteuccia struthiopteris*-treated groups, respectively. (**c**) Shows a boxplot of representative important factors that can be applied on the network module. The upper panel and lower panel depict the results for 3’-CMP.2 and 3’-AMP.5, respectively. Blue, green, and purple boxes represent cellulose-, *P. aquilinum*-, and *M. struthiopteris*-treated groups, respectively. *** *p* < 0.001 calculated by the Holm method.

Although the impact of additional food intake was detected using fecal metabolite PCA, others indicated complex dispersion. It was suggested that the same composition microbiota shifted to another state of metabolism or that treated mice responses of food intake were masked by other factors, such as mouse-specific characteristics and stress [22]. We managed to mine the data on the impact of food by machine learning using Random Forest, which was applied to all datasets and was used to create a multi-dimensional plot, indicating that each group had a specific factor that separated it from the others (Figure 3a, Figures S8a and S9a); we then selected the factors important for this separation, with careful cross-validation, to avoid over-training, using the Random Forest package (Figure 3b, Figures S8b and S9b). Random Forest revealed differences that were undetectable on PCA. The representative factors were clustered in the functional network module of the following network analysis (Figure 3c, Figures S8c and S9c). In addition, urinary ICP-OES data could not be separated using Random Forest analysis. These results indicate that urinary elements were not affected by food intake in this case.

### 3.3. Application of Important Factors to Network Modules for Separation

**Figure 4:**
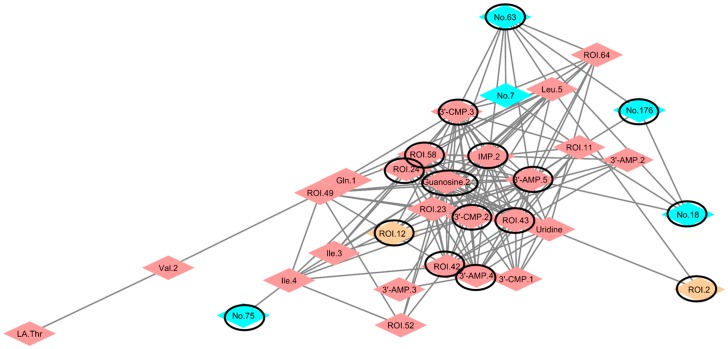
Purine/pyrimidine network with important bacteria and urinary metabolites of *Matteuccia struthiopteris*-treated mice based on the combined data set. The depicted network is based on calculations using the igraph package with Cytoscape. Blue, red, and light-yellow diamonds represent bacteria, fecal metabolites, and urinary metabolites, respectively (Tables S2 and S3). Black-line-circled diamonds represent factors selected by Random Forest as factors important for separating the groups.

We were able to detect important variables from the multivariate data by Random Forest and then attempted to find important variables in the network modules concerning the impact of additional food intake. We calculated the Spearman rank correlation coefficient with the combined dataset of the group treated with both *Pteridium aquilinum* and *Matteuccia struthiopteris* (metabolites of urine and feces and microbiome), performed analyses based on the correlation coefficients, using the R [45] package “igraph”, and then described the network community, using Cytoscape (Figure 4, Figures S10 and S11). The purine/pyrimidine network of *M. struthiopteris* indicated that the impact of *M. struthiopteris* intake affected the microbiota, metabolites, and host metabolism. Dietary purine/pyrimidine was important to avoid an allergic state and to develop gastrointestinal systems [53,54]. The bacterium that was an important factor in the *M. struthiopteris*-treated group, from the genus-associated purine/pyrimidine metabolism community, was *Prevotella*, which is a gram-negative bacterium, reported to be associated with fiber [15]. In addition, the genus *Akkermansia*, which is known to affect metabolism and reduce obesity and inflammation [55,56], responded to *M. struthiopteris* intake. Another community, including glucose signal-associated bacterium, was described (Figures S10b and S11c); however, important bacterium and important glucose signals were indirectly connected. Furthermore, the community did not associate with urinary metabolites. The results suggested that the impact of *M. struthiopteris* intake affects the purine/pyrimidine network more than glucose network. The important community and its associated bacterium *Akkermansia* were reported to be useful for health, so the impact of *M. struthiopteris* intake may involve a prebiotic-like effect of shifting nucleotide availability. Our method, involving a combination of machine learning and network visualization, detected a less significant food impact, which may help in the identification of prebiotics and probiotics that could be useful in the daily diet.

## 4. Conclusions

In this paper, we selected two pteridophytes, which may have led to varied nutrient compositions between land plants and seaweed, as targets to study the impact of food on the gut ecosystem in mice. These pteridophytes may be beneficial prebiotics in the daily diet because of their easy availability. We proposed a novel methodology for exploring the impact of food on the gut ecosystem based on a combination of machine learning and network visualization. We obtained total of 490 variables by multiple measurements from non-invasive, time-course sampled urine and feces, and finally detected two important network modules, based on 30 selected variables of importance, based on Random Forest calculations. This novel analytical method could detect the impact of a small, but significant, shift associated with *Matteuccia struthiopteris* intake, using the functional purine/pyrimidine network module.

## Supplementary Materials

The following are available online at www.mdpi.com/2072-6643/9/12/1307/s1. Figure S1: Tree diagram of plant components based on data set obtained by NMR measurement, Figure S2: Flow chart of the analysis procedure in this study, Figure S3: ^1^H-^13^C HSQC NMR spectra of *Pteridium aquilinum* and *Matteuccia struthiopteris* extracted by KPi/D_2_O solvent, Figure S4: ^1^H-^13^C HSQC NMR spectra of *Pteridium aquilinum* and *Matteuccia struthiopteris* components extracted by DMSO-d_6_/pyridine-d_5_ (4:1) solvent, Figure S5: Annotation of metabolites in urine NMR spectra, Figure S6: Annotation of metabolites in fecal NMR spectra, Figure S7: Principal component analysis of urinary metabolites and ions, Figure S8: The results of Random Forest for the fecal microbiome, Figure S9: The results of Random Forest for urinary metabolites, Figure S10: Network community correlation coefficients for *Matteuccia struthiopteris*-treated mice based on the combined data set, Figure S11: Network community correlation coefficients for *Pteridium aquilinum*-treated mice based on the combined data set, Table S1: Information on primers used for performing microbiome analysis, Table S2: List of sequential numbers for bacterial detection by MiSeq analysis, Table S3: List of annotated metabolites and unknown signals from NMR measurements.
