# Supplementary material for: Exploring the Impact of Food on the Gut Ecosystem Based on the Combination of Machine Learning and Network Visualization
Source: Nutrients. 2017 Dec 1;9(12):1307. doi: 10.3390/nu9121307 (PMC5748757; doi:10.3390/nu9121307)
Supplement: Supplementary file 1 [file nutrients-09-01307-s001.pdf]

nutrients

Supplementary Materials:

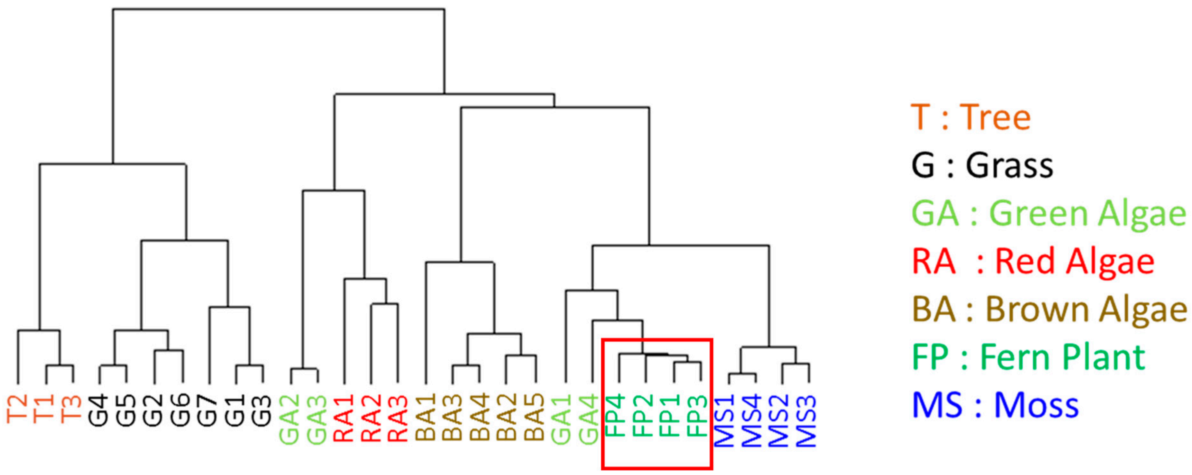

Figure S1. Tree diagram of plant components based on data set obtained by NMR measurement. This image is a modified version of a tree diagram. Raw or boiled edible parts of *Pteridium aquilinum* and *Matteuccia struthiopteris* classified at an intermediate point between moss and algae, not in higher plants, are surrounded by a red line. This image was partially revised from original presented in [31].

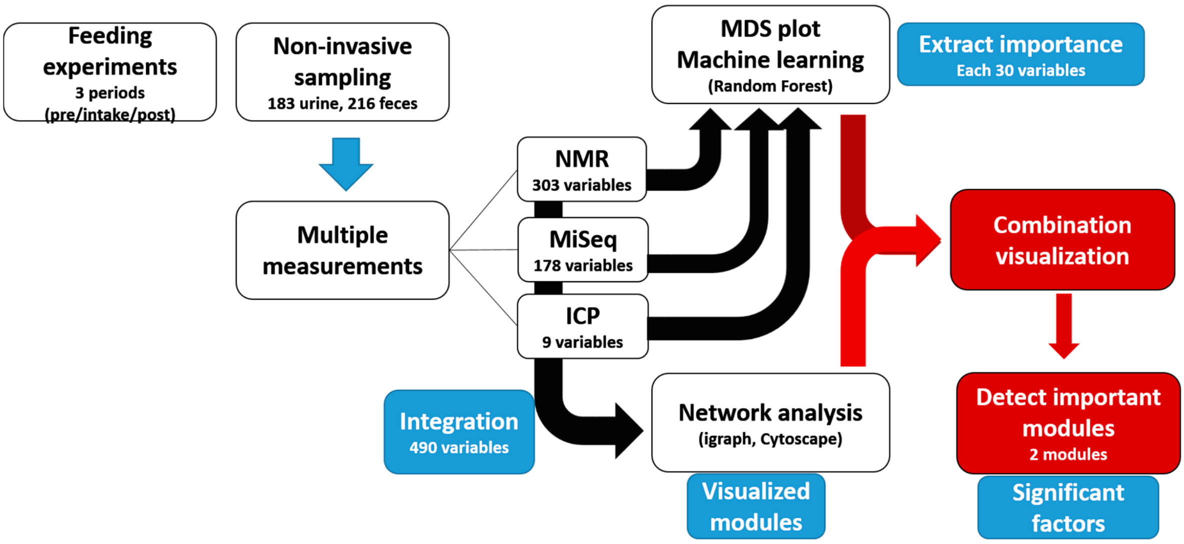

Figure S2. Flow chart of the analytical procedure in this study.

This flow chart indicates analytical steps. Arrows indicated experimental or analytical direction. Black arrows meant already exist methods or analysis steps. Text in the blue boxes show concept of the steps. Red arrows and box meant a novel approach developed in this study. This study shows that detection method of significant factor which respond to food intake at the final step.

Table S1. Information on primers used for performing microbiome analysis.

name	sequence
v4.SA501	AATGATACGGCGACCACCGAGATCTACACATCGTACGTATGGTAATTGTGTGCCAGCMGCCGCGGTAA
v4.SA502	AATGATACGGCGACCACCGAGATCTACACACTATCTGTATGGTAATTGTGTGCCAGCMGCCGCGGTAA
v4.SA503	AATGATACGGCGACCACCGAGATCTACACTAGCGAGTTATGGTAATTGTGTGCCAGCMGCCGCGGTAA
v4.SA504	AATGATACGGCGACCACCGAGATCTACACCTGCGTGTTATGGTAATTGTGTGCCAGCMGCCGCGGTAA
v4.SA505	AATGATACGGCGACCACCGAGATCTACACTCATCGAGTATGGTAATTGTGTGCCAGCMGCCGCGGTAA
v4.SA506	AATGATACGGCGACCACCGAGATCTACACCGTGAGTGTATGGTAATTGTGTGCCAGCMGCCGCG GTAA
v4.SA507	AATGATACGGCGACCACCGAGATCTACACGGATATCTTATGGTAATTGTGTGCCAGCMGCCGCGGTAA
v4.SA508	AATGATACGGCGACCACCGAGATCTACACGACACCGTTATGGTAATTGTGTGCCAGCMGCCGCGGTAA
v4.SB501	AATGATACGGCGACCACCGAGATCTACACCTACTATATATGGTAATTGTGTGCCAGCMGCCGCGGTAA
v4.SB502	AATGATACGGCGACCACCGAGATCTACACCGTTACTATATGGTAATTGTGTGCCAGCMGCCGCGGTAA
v4.SB503	AATGATACGGCGACCACCGAGATCTACACAGAGTCACTATGGTAATTGTGTGCCAGCMGCCGCGGTAA
v4.SB504	AATGATACGGCGACCACCGAGATCTACACTACGAGACTATGGTAATTGTGTGCCAGCMGCCGCGGTAA
v4.SB505	AATGATACGGCGACCACCGAGATCTACACACGTCTCGTATGGTAATTGTGTGCCAGCMGCCGCGGTAA
v4.SB506	AATGATACGGCGACCACCGAGATCTACACTCGACGAGTATGGTAATTGTGTGCCAGCMGCCGCGGTAA
v4.SB507	AATGATACGGCGACCACCGAGATCTACACGATCGTGTTATGGTAATTGTGTGCCAGCMGCCGCGGTAA
v4.SB508	AATGATACGGCGACCACCGAGATCTACACGTCAGATATATGGTAATTGTGTGCCAGCMGCCGCGGTAA
v4.SA701	CAAGCAGAAGACGGCATACGAGATAACTCTCGAGTCAGTCAGCCGGACTACHVGGGTWTCTAAT
v4.SA702	CAAGCAGAAGACGGCATACGAGATACTATGTCAGTCAGTCAGCCGGACTACHVGGGTWTCTAAT
v4.SA703	CAAGCAGAAGACGGCATACGAGATAGTAGCGTAGTCAGTCAGCCGGACTACHVGGGTWTCTAAT
v4.SA704	CAAGCAGAAGACGGCATACGAGATCAGTGAGTAGTCAGTCAGCCGGACTACHVGGGTWTCTAAT
v4.SA705	CAAGCAGAAGACGGCATACGAGATCGTACTCAAGTCAGTCAGCCGGACTACHVGGGTWTCTAAT
v4.SA706	CAAGCAGAAGACGGCATACGAGATCTACGCAGAGTCAGTCAGCCGGACTACHVGGGTWTCTAAT
v4.SA707	CAAGCAGAAGACGGCATACGAGATGGAGACTAAGTCAGTCAGCCGGACTACHVGGGTWTCTAAT
v4.SA708	CAAGCAGAAGACGGCATACGAGATGTCGCTCGAGTCAGTCAGCCGGACTACHVGGGTWTCTAAT
v4.SA709	CAAGCAGAAGACGGCATACGAGATGTCGTAGTAGTCAGTCAGCCGGACTACHVGGGTWTCTAAT
v4.SA710	CAAGCAGAAGACGGCATACGAGATTAGCAGACAGTCAGTCAGCCGGACTACHVGGGTWTCTAAT
v4.SA711	CAAGCAGAAGACGGCATACGAGATTCATAGACAGTCAGTCAGCCGGACTACHVGGGTWTCTAAT
v4.SA712	CAAGCAGAAGACGGCATACGAGATTCGCTATAAGTCAGTCAGCCGGACTACHVGGGTWTCTAAT
v4.SB701	CAAGCAGAAGACGGCATACGAGATAAGTCGAGAGTCAGTCAGCCGGACTACHVGGGTWTCTAAT
v4.SB702	CAAGCAGAAGACGGCATACGAGATATACTTCGAGTCAGTCAGCCGGACTACHVGGGTWTCTAAT
v4.SB703	CAAGCAGAAGACGGCATACGAGATAGCTGCTAAGTCAGTCAGCCGGACTACHVGGGTWTCTAAT
v4.SB704	CAAGCAGAAGACGGCATACGAGATCATAGAGAAGTCAGTCAGCCGGACTACHVGGGTWTCTAAT
v4.SB705	CAAGCAGAAGACGGCATACGAGATCGTAGATCAGTCAGTCAGCCGGACTACHVGGGTWTCTAAT
v4.SB706	CAAGCAGAAGACGGCATACGAGATCTCGTTACAGTCAGTCAGCCGGACTACHVGGGTWTCTAAT
v4.SB707	CAAGCAGAAGACGGCATACGAGATGCGCACGTAGTCAGTCAGCCGGACTACHVGGGTWTCTAAT
v4.SB708	CAAGCAGAAGACGGCATACGAGATGGTACTATAGTCAGTCAGCCGGACTACHVGGGTWTCTAAT
v4.SB709	CAAGCAGAAGACGGCATACGAGATGTATACGCAGTCAGTCAGCCGGACTACHVGGGTWTCTAAT
v4.SB710	CAAGCAGAAGACGGCATACGAGATTACGAGCAAGTCAGTCAGCCGGACTACHVGGGTWTCTAAT
v4.SB711	CAAGCAGAAGACGGCATACGAGATTCAGCGTTAGTCAGTCAGCCGGACTACHVGGGTWTCTAAT
v4.SB712	CAAGCAGAAGACGGCATACGAGATTCGCTACGAGTCAGTCAGCCGGACTACHVGGGTWTCTAAT
v4.SB501	AATGATACGGCGACCACCGAGATCTACACCTACTATATATGGTAATTGTGTGCCAGCMGCCGCGGTAA
v4.SB502	AATGATACGGCGACCACCGAGATCTACACCGTTACTATATGGTAATTGTGTGCCAGCMGCCGCGGTAA
v4.SB503	AATGATACGGCGACCACCGAGATCTACACAGAGTCACTATGGTAATTGTGTGCCAGCMGCCGCGGTAA
v4.SB504	AATGATACGGCGACCACCGAGATCTACACTACGAGACTATGGTAATTGTGTGCCAGCMGCCGCGGTAA
v4.SB505	AATGATACGGCGACCACCGAGATCTACACACGTCTCGTATGGTAATTGTGTGCCAGCMGCCGCGGTAA
v4.SB506	AATGATACGGCGACCACCGAGATCTACACTCGACGAGTATGGTAATTGTGTGCCAGCMGCCGCGGTAA
v4.SB507	AATGATACGGCGACCACCGAGATCTACACGATCGTGTTATGGTAATTGTGTGCCAGCMGCCGCGGTAA
v4.SB508	AATGATACGGCGACCACCGAGATCTACACGTCAGATATATGGTAATTGTGTGCCAGCMGCCGCGGTAA
v4.SB701	CAAGCAGAAGACGGCATACGAGATAAGTCGAGAGTCAGTCAGCCGGACTACHVGGGTWTCTAAT
v4.SB702	CAAGCAGAAGACGGCATACGAGATATACTTCGAGTCAGTCAGCCGGACTACHVGGGTWTCTAAT
v4.SB703	CAAGCAGAAGACGGCATACGAGATAGCTGCTAAGTCAGTCAGCCGGACTACHVGGGTWTCTAAT
v4.SB704	CAAGCAGAAGACGGCATACGAGATCATAGAGAAGTCAGTCAGCCGGACTACHVGGGTWTCTAAT
v4.SB705	CAAGCAGAAGACGGCATACGAGATCGTAGATCAGTCAGTCAGCCGGACTACHVGGGTWTCTAAT
v4.SB706	CAAGCAGAAGACGGCATACGAGATCTCGTTACAGTCAGTCAGCCGGACTACHVGGGTWTCTAAT
v4.SB707	CAAGCAGAAGACGGCATACGAGATGCGCACGTAGTCAGTCAGCCGGACTACHVGGGTWTCTAAT
v4.SB708	CAAGCAGAAGACGGCATACGAGATGGTACTATAGTCAGTCAGCCGGACTACHVGGGTWTCTAAT
v4.SB709	CAAGCAGAAGACGGCATACGAGATGTATACGCAGTCAGTCAGCCGGACTACHVGGGTWTCTAAT
v4.SB710	CAAGCAGAAGACGGCATACGAGATTACGAGCAAGTCAGTCAGCCGGACTACHVGGGTWTCTAAT
v4.SB711	CAAGCAGAAGACGGCATACGAGATTCAGCGTTAGTCAGTCAGCCGGACTACHVGGGTWTCTAAT
v4.SB712	CAAGCAGAAGACGGCATACGAGATTCGCTACGAGTCAGTCAGCCGGACTACHVGGGTWTCTAAT

This primer information is referenced from a previous report [41].

Table S2. List of sequential numbers for bacterial detection by MiSeq analysis. Numbers in all Figures are equivalent to bacterial names in this list.

No.1	k__Bacteria;Other;Other;Other;Other;Other
No.2	k__Bacteria;p__Actinobacteria;Other;Other;Other;Other
No.3	k__Bacteria;p__Actinobacteria;c__Actinobacteria;o__Actinomycetales;f__Brevibacteriaceae;g__Brevibacterium
No.4	k__Bacteria;p__Actinobacteria;c__Actinobacteria;o__Actinomycetales;f__Corynebacteriaceae;g__Corynebacterium
No.5	k__Bacteria;p__Actinobacteria;c__Actinobacteria;o__Actinomycetales;f__Geodermatophilaceae;Other
No.6	k__Bacteria;p__Actinobacteria;c__Actinobacteria;o__Actinomycetales;f__Micrococcaceae;g__Kocuria
No.7	k__Bacteria;p__Actinobacteria;c__Actinobacteria;o__Actinomycetales;f__Micrococcaceae;g__Renibacterium
No.8	k__Bacteria;p__Actinobacteria;c__Actinobacteria;o__Actinomycetales;f__Mycobacteriaceae;g__Mycobacterium
No.9	k__Bacteria;p__Actinobacteria;c__Actinobacteria;o__Actinomycetales;f__Pseudonocardiaceae;Other
No.10	k__Bacteria;p__Actinobacteria;c__Coriobacteriia;o__Coriobacteriales;f__Coriobacteriaceae;Other
No.11	k__Bacteria;p__Actinobacteria;c__Coriobacteriia;o__Coriobacteriales;f__Coriobacteriaceae;g__
No.12	k__Bacteria;p__Actinobacteria;c__Coriobacteriia;o__Coriobacteriales;f__Coriobacteriaceae;g__Adlercreutzia
No.13	k__Bacteria;p__Actinobacteria;c__Thermoleophilia;o__Gaiellales;f__AK1AB1_02E;g__
No.14	k__Bacteria;p__Bacteroidetes;Other;Other;Other;Other
No.15	k__Bacteria;p__Bacteroidetes;c__Bacteroidia;o__Bacteroidales;Other;Other
No.16	k__Bacteria;p__Bacteroidetes;c__Bacteroidia;o__Bacteroidales;f__Porphyromonadaceae;g__Parabacteroides
No.17	k__Bacteria;p__Bacteroidetes;c__Bacteroidia;o__Bacteroidales;f__Prevotellaceae;Other
No.18	k__Bacteria;p__Bacteroidetes;c__Bacteroidia;o__Bacteroidales;f__Prevotellaceae;g__Prevotella
No.19	k__Bacteria;p__Bacteroidetes;c__Bacteroidia;o__Bacteroidales;f__Rikenellaceae;Other
No.20	k__Bacteria;p__Bacteroidetes;c__Bacteroidia;o__Bacteroidales;f__Rikenellaceae;g__
No.21	k__Bacteria;p__Bacteroidetes;c__Bacteroidia;o__Bacteroidales;f__S24-7;g__
No.22	k__Bacteria;p__Bacteroidetes;c__Bacteroidia;o__Bacteroidales;f__[Barnesiellaceae];g__
No.23	k__Bacteria;p__Bacteroidetes;c__Flavobacteriia;o__Flavobacteriales;Other;Other
No.24	k__Bacteria;p__Bacteroidetes;c__Flavobacteriia;o__Flavobacteriales;f__;g__
No.25	k__Bacteria;p__Bacteroidetes;c__Flavobacteriia;o__Flavobacteriales;f__Cryomorphaceae;g__Cryomorpha
No.26	k__Bacteria;p__Bacteroidetes;c__Flavobacteriia;o__Flavobacteriales;f__Flavobacteriaceae;Other
No.27	k__Bacteria;p__Bacteroidetes;c__Flavobacteriia;o__Flavobacteriales;f__Flavobacteriaceae;g__Ascidianibacter
No.28	k__Bacteria;p__Bacteroidetes;c__Flavobacteriia;o__Flavobacteriales;f__Flavobacteriaceae;g__Polaribacter
No.29	k__Bacteria;p__Bacteroidetes;c__Flavobacteriia;o__Flavobacteriales;f__Flavobacteriaceae;g__Sediminicola
No.30	k__Bacteria;p__Bacteroidetes;c__Flavobacteriia;o__Flavobacteriales;f__[Weeksellaceae];g__Chryseobacterium
No.31	k__Bacteria;p__Bacteroidetes;c__Flavobacteriia;o__Flavobacteriales;f__[Weeksellaceae];g__Cloacibacterium
No.32	k__Bacteria;p__Bacteroidetes;c__[Rhodothermi];o__[Rhodothermales];f__Rhodothermaceae;g__
No.33	k__Bacteria;p__Bacteroidetes;c__[Saprospirae];o__[Saprospirales];f__Chitinophagaceae;g__Lacibacter
No.34	k__Bacteria;p__Bacteroidetes;c__[Saprospirae];o__[Saprospirales];f__Chitinophagaceae;g__Sediminibacterium
No.35	k__Bacteria;p__Bacteroidetes;c__[Saprospirae];o__[Saprospirales];f__Saprospiraceae;g__
No.36	k__Bacteria;p__Chlamydiae;c__Chlamydiia;o__Chlamydiales;Other;Other
No.37	k__Bacteria;p__Chlamydiae;c__Chlamydiia;o__Chlamydiales;f__;g__
No.38	k__Bacteria;p__Cyanobacteria;Other;Other;Other;Other
No.39	k__Bacteria;p__Cyanobacteria;c__4C0d-2;o__YS2;f__;g__
No.40	k__Bacteria;p__Cyanobacteria;c__Chloroplast;o__Stramenopiles;f__;g__
No.41	k__Bacteria;p__Cyanobacteria;c__Chloroplast;o__Streptophyta;f__;g__
No.42	k__Bacteria;p__Cyanobacteria;c__Oscillatoriophycideae;Other;Other;Other
No.43	k__Bacteria;p__Cyanobacteria;c__Oscillatoriophycideae;o__Chroococcales;f__Xenococcaceae;g__
No.44	k__Bacteria;p__Deferribacteres;c__Deferribacteres;o__Deferribacterales;f__Deferribacteraceae;g__Mucispirillum
No.45	k__Bacteria;p__Firmicutes;Other;Other;Other;Other
No.46	k__Bacteria;p__Firmicutes;c__Bacilli;Other;Other;Other
No.47	k__Bacteria;p__Firmicutes;c__Bacilli;o__Bacillales;f__Bacillaceae;g__Bacillus
No.48	k__Bacteria;p__Firmicutes;c__Bacilli;o__Bacillales;f__Bacillaceae;g__Oceanobacillus
No.49	k__Bacteria;p__Firmicutes;c__Bacilli;o__Bacillales;f__Staphylococcaceae;g__Jeotgalicoccus
No.50	k__Bacteria;p__Firmicutes;c__Bacilli;o__Bacillales;f__Staphylococcaceae;g__Staphylococcus

Table S2. (Continued)

No.51	k__Bacteria;p__Firmicutes;c__Bacilli;o__Lactobacillales;Other;Other
No.52	k__Bacteria;p__Firmicutes;c__Bacilli;o__Lactobacillales;f__Aerococcaceae;g__
No.53	k__Bacteria;p__Firmicutes;c__Bacilli;o__Lactobacillales;f__Enterococcaceae;Other
No.54	k__Bacteria;p__Firmicutes;c__Bacilli;o__Lactobacillales;f__Lactobacillaceae;Other
No.55	k__Bacteria;p__Firmicutes;c__Bacilli;o__Lactobacillales;f__Lactobacillaceae;g__Lactobacillus
No.56	k__Bacteria;p__Firmicutes;c__Bacilli;o__Lactobacillales;f__Leuconostocaceae;Other
No.57	k__Bacteria;p__Firmicutes;c__Bacilli;o__Lactobacillales;f__Streptococcaceae;g__Lactococcus
No.58	k__Bacteria;p__Firmicutes;c__Bacilli;o__Lactobacillales;f__Streptococcaceae;g__Streptococcus
No.59	k__Bacteria;p__Firmicutes;c__Bacilli;o__Turicibacterales;f__Turicibacteraceae;g__Turicibacter
No.60	k__Bacteria;p__Firmicutes;c__Clostridia;Other;Other;Other
No.61	k__Bacteria;p__Firmicutes;c__Clostridia;o__Clostridiales;Other;Other
No.62	k__Bacteria;p__Firmicutes;c__Clostridia;o__Clostridiales;f__;g__
No.63	k__Bacteria;p__Firmicutes;c__Clostridia;o__Clostridiales;f__Clostridiaceae;Other
No.64	k__Bacteria;p__Firmicutes;c__Clostridia;o__Clostridiales;f__Clostridiaceae;g__Clostridium
No.65	k__Bacteria;p__Firmicutes;c__Clostridia;o__Clostridiales;f__Dehalobacteriaceae;g__Dehalobacterium
No.66	k__Bacteria;p__Firmicutes;c__Clostridia;o__Clostridiales;f__Eubacteriaceae;g__Anaerofustis
No.67	k__Bacteria;p__Firmicutes;c__Clostridia;o__Clostridiales;f__Lachnospiraceae;Other
No.68	k__Bacteria;p__Firmicutes;c__Clostridia;o__Clostridiales;f__Lachnospiraceae;g__
No.69	k__Bacteria;p__Firmicutes;c__Clostridia;o__Clostridiales;f__Lachnospiraceae;g__Anaerostipes
No.70	k__Bacteria;p__Firmicutes;c__Clostridia;o__Clostridiales;f__Lachnospiraceae;g__Blautia
No.71	k__Bacteria;p__Firmicutes;c__Clostridia;o__Clostridiales;f__Lachnospiraceae;g__Clostridium
No.72	k__Bacteria;p__Firmicutes;c__Clostridia;o__Clostridiales;f__Lachnospiraceae;g__Coprococcus
No.73	k__Bacteria;p__Firmicutes;c__Clostridia;o__Clostridiales;f__Lachnospiraceae;g__Dorea
No.74	k__Bacteria;p__Firmicutes;c__Clostridia;o__Clostridiales;f__Lachnospiraceae;g__Epulopiscium
No.75	k__Bacteria;p__Firmicutes;c__Clostridia;o__Clostridiales;f__Lachnospiraceae;g__Roseburia
No.76	k__Bacteria;p__Firmicutes;c__Clostridia;o__Clostridiales;f__Lachnospiraceae;g__[Ruminococcus]
No.77	k__Bacteria;p__Firmicutes;c__Clostridia;o__Clostridiales;f__Peptococcaceae;g__
No.78	k__Bacteria;p__Firmicutes;c__Clostridia;o__Clostridiales;f__Peptostreptococcaceae;Other
No.79	k__Bacteria;p__Firmicutes;c__Clostridia;o__Clostridiales;f__Ruminococcaceae;Other
No.80	k__Bacteria;p__Firmicutes;c__Clostridia;o__Clostridiales;f__Ruminococcaceae;g__
No.81	k__Bacteria;p__Firmicutes;c__Clostridia;o__Clostridiales;f__Ruminococcaceae;g__Anaerotruncus
No.82	k__Bacteria;p__Firmicutes;c__Clostridia;o__Clostridiales;f__Ruminococcaceae;g__Butyricicoccus
No.83	k__Bacteria;p__Firmicutes;c__Clostridia;o__Clostridiales;f__Ruminococcaceae;g__Clostridium
No.84	k__Bacteria;p__Firmicutes;c__Clostridia;o__Clostridiales;f__Ruminococcaceae;g__Faecalibacterium
No.85	k__Bacteria;p__Firmicutes;c__Clostridia;o__Clostridiales;f__Ruminococcaceae;g__Oscillospira
No.86	k__Bacteria;p__Firmicutes;c__Clostridia;o__Clostridiales;f__Ruminococcaceae;g__Ruminococcus
No.87	k__Bacteria;p__Firmicutes;c__Clostridia;o__Clostridiales;f__Veillonellaceae;g__Veillonella
No.88	k__Bacteria;p__Firmicutes;c__Clostridia;o__Clostridiales;f__[Mogibacteriaceae];Other
No.89	k__Bacteria;p__Firmicutes;c__Clostridia;o__Clostridiales;f__[Mogibacteriaceae];g__
No.90	k__Bacteria;p__Firmicutes;c__Clostridia;o__Clostridiales;f__[Tissierellaceae];g__Anaerococcus
No.91	k__Bacteria;p__Firmicutes;c__Erysipelotrichi;o__Erysipelotrichales;f__Erysipelotrichaceae;Other
No.92	k__Bacteria;p__Firmicutes;c__Erysipelotrichi;o__Erysipelotrichales;f__Erysipelotrichaceae;g__
No.93	k__Bacteria;p__Firmicutes;c__Erysipelotrichi;o__Erysipelotrichales;f__Erysipelotrichaceae;g__Allobaculum
No.94	k__Bacteria;p__Firmicutes;c__Erysipelotrichi;o__Erysipelotrichales;f__Erysipelotrichaceae;g__Clostridium
No.95	k__Bacteria;p__Firmicutes;c__Erysipelotrichi;o__Erysipelotrichales;f__Erysipelotrichaceae;g__Coprobacillus
No.96	k__Bacteria;p__Firmicutes;c__Erysipelotrichi;o__Erysipelotrichales;f__Erysipelotrichaceae;g__Erysipelothrix
No.97	k__Bacteria;p__Fusobacteria;c__Fusobacteriia;o__Fusobacteriales;f__Fusobacteriaceae;g__Cetobacterium
No.98	k__Bacteria;p__Fusobacteria;c__Fusobacteriia;o__Fusobacteriales;f__Leptotrichiaceae;g__Leptotrichia
No.99	k__Bacteria;p__Lentisphaerae;c__[Lentisphaeria];o__Lentisphaerales;f__Lentisphaeraceae;g__
No.100	k__Bacteria;p__Planctomycetes;c__OM190;o__agg27;f__;g__

Table S2. (Continued)

No.101	k__Bacteria;p__Planctomycetes;c__Planctomycetia;o__Gemmatales;f__Gemmataceae;g__
No.102	k__Bacteria;p__Planctomycetes;c__Planctomycetia;o__Pirellulales;f__Pirellulaceae;g__
No.103	k__Bacteria;p__Proteobacteria;Other;Other;Other;Other
No.104	k__Bacteria;p__Proteobacteria;c__Alphaproteobacteria;Other;Other;Other
No.105	k__Bacteria;p__Proteobacteria;c__Alphaproteobacteria;o__Caulobacterales;f__Caulobacteraceae;Other
No.106	k__Bacteria;p__Proteobacteria;c__Alphaproteobacteria;o__Rhizobiales;Other;Other
No.107	k__Bacteria;p__Proteobacteria;c__Alphaproteobacteria;o__Rhizobiales;f__Bradyrhizobiaceae;Other
No.108	k__Bacteria;p__Proteobacteria;c__Alphaproteobacteria;o__Rhizobiales;f__Bradyrhizobiaceae;g__Balneimonas
No.109	k__Bacteria;p__Proteobacteria;c__Alphaproteobacteria;o__Rhizobiales;f__Bradyrhizobiaceae;g__Bradyrhizobium
No.110	k__Bacteria;p__Proteobacteria;c__Alphaproteobacteria;o__Rhizobiales;f__Hyphomicrobiaceae;Other
No.111	k__Bacteria;p__Proteobacteria;c__Alphaproteobacteria;o__Rhizobiales;f__Methylobacteriaceae;g__
No.112	k__Bacteria;p__Proteobacteria;c__Alphaproteobacteria;o__Rhizobiales;f__Methylobacteriaceae;g__Methylobacterium
No.113	k__Bacteria;p__Proteobacteria;c__Alphaproteobacteria;o__Rhizobiales;f__Phyllobacteriaceae;Other
No.114	k__Bacteria;p__Proteobacteria;c__Alphaproteobacteria;o__Rhizobiales;f__Phyllobacteriaceae;g__Phyllobacterium
No.115	k__Bacteria;p__Proteobacteria;c__Alphaproteobacteria;o__Rhizobiales;f__Rhizobiaceae;Other
No.116	k__Bacteria;p__Proteobacteria;c__Alphaproteobacteria;o__Rhizobiales;f__Xanthobacteraceae;g__
No.117	k__Bacteria;p__Proteobacteria;c__Alphaproteobacteria;o__Rhodobacterales;f__Rhodobacteraceae;Other
No.118	k__Bacteria;p__Proteobacteria;c__Alphaproteobacteria;o__Rhodobacterales;f__Rhodobacteraceae;g__Loktanella
No.119	k__Bacteria;p__Proteobacteria;c__Alphaproteobacteria;o__Rhodobacterales;f__Rhodobacteraceae;g__Paracoccus
No.120	k__Bacteria;p__Proteobacteria;c__Alphaproteobacteria;o__Rhodobacterales;f__Rhodobacteraceae;g__Phaeobacter
No.121	k__Bacteria;p__Proteobacteria;c__Alphaproteobacteria;o__Rhodobacterales;f__Rhodobacteraceae;g__Rhodobacter
No.122	k__Bacteria;p__Proteobacteria;c__Alphaproteobacteria;o__Rhodospirillales;f__Acetobacteraceae;Other
No.123	k__Bacteria;p__Proteobacteria;c__Alphaproteobacteria;o__Rhodospirillales;f__Acetobacteraceae;g__Roseomonas
No.124	k__Bacteria;p__Proteobacteria;c__Alphaproteobacteria;o__Rhodospirillales;f__Rhodospirillaceae;Other
No.125	k__Bacteria;p__Proteobacteria;c__Alphaproteobacteria;o__Rickettsiales;f__mitochondria;Other
No.126	k__Bacteria;p__Proteobacteria;c__Alphaproteobacteria;o__Sphingomonadales;f__Erythrobacteraceae;Other
No.127	k__Bacteria;p__Proteobacteria;c__Alphaproteobacteria;o__Sphingomonadales;f__Sphingomonadaceae;Other
No.128	k__Bacteria;p__Proteobacteria;c__Alphaproteobacteria;o__Sphingomonadales;f__Sphingomonadaceae;g__Sphingomonas
No.129	k__Bacteria;p__Proteobacteria;c__Betaproteobacteria;o__Burkholderiales;f__Alcaligenaceae;g__Achromobacter
No.130	k__Bacteria;p__Proteobacteria;c__Betaproteobacteria;o__Burkholderiales;f__Comamonadaceae;Other
No.131	k__Bacteria;p__Proteobacteria;c__Betaproteobacteria;o__Burkholderiales;f__Comamonadaceae;g__Comamonas
No.132	k__Bacteria;p__Proteobacteria;c__Betaproteobacteria;o__Burkholderiales;f__Comamonadaceae;g__Tepidimonas
No.133	k__Bacteria;p__Proteobacteria;c__Betaproteobacteria;o__Burkholderiales;f__Oxalobacteraceae;Other
No.134	k__Bacteria;p__Proteobacteria;c__Betaproteobacteria;o__Rhodocyclales;f__Rhodocyclaceae;Other
No.135	k__Bacteria;p__Proteobacteria;c__Deltaproteobacteria;o__Desulfovibrionales;f__Desulfovibrionaceae;g__
No.136	k__Bacteria;p__Proteobacteria;c__Deltaproteobacteria;o__Desulfovibrionales;f__Desulfovibrionaceae;g__Desulfovibrio
No.137	k__Bacteria;p__Proteobacteria;c__Gammaproteobacteria;Other;Other;Other
No.138	k__Bacteria;p__Proteobacteria;c__Gammaproteobacteria;o__Alteromonadales;Other;Other
No.139	k__Bacteria;p__Proteobacteria;c__Gammaproteobacteria;o__Alteromonadales;f__Alteromonadaceae;g__Alteromonas
No.140	k__Bacteria;p__Proteobacteria;c__Gammaproteobacteria;o__Alteromonadales;f__Alteromonadaceae;g__BD2-13
No.141	k__Bacteria;p__Proteobacteria;c__Gammaproteobacteria;o__Alteromonadales;f__Alteromonadaceae;g__Marinobacter
No.142	k__Bacteria;p__Proteobacteria;c__Gammaproteobacteria;o__Alteromonadales;f__Colwelliaceae;Other
No.143	k__Bacteria;p__Proteobacteria;c__Gammaproteobacteria;o__Alteromonadales;f__Colwelliaceae;g__
No.144	k__Bacteria;p__Proteobacteria;c__Gammaproteobacteria;o__Alteromonadales;f__Idiomarinaceae;g__Idiomarina
No.145	k__Bacteria;p__Proteobacteria;c__Gammaproteobacteria;o__Alteromonadales;f__OM60;g__
No.146	k__Bacteria;p__Proteobacteria;c__Gammaproteobacteria;o__Alteromonadales;f__Shewanellaceae;g__Shewanella
No.147	k__Bacteria;p__Proteobacteria;c__Gammaproteobacteria;o__Enterobacteriales;f__Enterobacteriaceae;Other
No.148	k__Bacteria;p__Proteobacteria;c__Gammaproteobacteria;o__Enterobacteriales;f__Enterobacteriaceae;g__Serratia
No.149	k__Bacteria;p__Proteobacteria;c__Gammaproteobacteria;o__HTCC2188;f__HTCC2089;g__
No.150	k__Bacteria;p__Proteobacteria;c__Gammaproteobacteria;o__Legionellales;f__Legionellaceae;Other

Table S2. (Continued)

No.151	k__Bacteria;p__Proteobacteria;c__Gammaproteobacteria;o__Legionellales;f__Legionellaceae;g__
No.152	k__Bacteria;p__Proteobacteria;c__Gammaproteobacteria;o__Oceanospirillales;f__Alcanivoracaceae;g__Alcanivorax
No.153	k__Bacteria;p__Proteobacteria;c__Gammaproteobacteria;o__Oceanospirillales;f__Halomonadaceae;g__Halomonas
No.154	k__Bacteria;p__Proteobacteria;c__Gammaproteobacteria;o__Oceanospirillales;f__Oceanospirillaceae;g__Oleibacter
No.155	k__Bacteria;p__Proteobacteria;c__Gammaproteobacteria;o__Oceanospirillales;f__Oceanospirillaceae;g__Oleispira
No.156	k__Bacteria;p__Proteobacteria;c__Gammaproteobacteria;o__Oceanospirillales;f__Oceanospirillaceae;g__Spongiispira
No.157	k__Bacteria;p__Proteobacteria;c__Gammaproteobacteria;o__Pseudomonadales;f__Moraxellaceae;g__Acinetobacter
No.158	k__Bacteria;p__Proteobacteria;c__Gammaproteobacteria;o__Pseudomonadales;f__Moraxellaceae;g__Enhydrobacter
No.159	k__Bacteria;p__Proteobacteria;c__Gammaproteobacteria;o__Pseudomonadales;f__Pseudomonadaceae;Other
No.160	k__Bacteria;p__Proteobacteria;c__Gammaproteobacteria;o__Pseudomonadales;f__Pseudomonadaceae;g__Pseudomonas
No.161	k__Bacteria;p__Proteobacteria;c__Gammaproteobacteria;o__Thiotrichales;f__Piscirickettsiaceae;g__
No.162	k__Bacteria;p__Proteobacteria;c__Gammaproteobacteria;o__Vibrionales;f__Pseudoalteromonadaceae;g__Pseudoalteromonas
No.163	k__Bacteria;p__Proteobacteria;c__Gammaproteobacteria;o__Vibrionales;f__Vibrionaceae;Other
No.164	k__Bacteria;p__Proteobacteria;c__Gammaproteobacteria;o__Vibrionales;f__Vibrionaceae;g__Photobacterium
No.165	k__Bacteria;p__Proteobacteria;c__Gammaproteobacteria;o__Vibrionales;f__Vibrionaceae;g__Vibrio
No.166	k__Bacteria;p__Proteobacteria;c__Gammaproteobacteria;o__Xanthomonadales;f__Xanthomonadaceae;Other
No.167	k__Bacteria;p__Proteobacteria;c__Gammaproteobacteria;o__Xanthomonadales;f__Xanthomonadaceae;g__Stenotrophomonas
No.168	k__Bacteria;p__Proteobacteria;c__Gammaproteobacteria;o__Xanthomonadales;f__Xanthomonadaceae;g__Xanthomonas
No.169	k__Bacteria;p__Proteobacteria;c__Gammaproteobacteria;o__[Marinicellales];f__[Marinicellaceae];g__
No.170	k__Bacteria;p__Proteobacteria;c__Gammaproteobacteria;o__[Marinicellales];f__[Marinicellaceae];g__Marinicella
No.171	k__Bacteria;p__SBR1093;c__EC214;o__;f__;g__
No.172	k__Bacteria;p__Tenericutes;c__Mollicutes;o__Mycoplasmatales;f__Mycoplasmataceae;g__
No.173	k__Bacteria;p__Tenericutes;c__Mollicutes;o__Mycoplasmatales;f__Mycoplasmataceae;g__Mycoplasma
No.174	k__Bacteria;p__Tenericutes;c__Mollicutes;o__RF39;f__;g__
No.175	k__Bacteria;p__Verrucomicrobia;c__Verrucomicrobiae;o__Verrucomicrobiales;f__Verrucomicrobiaceae;g__
No.176	k__Bacteria;p__Verrucomicrobia;c__Verrucomicrobiae;o__Verrucomicrobiales;f__Verrucomicrobiaceae;g__Akkermansia
No.177	k__Bacteria;p__Verrucomicrobia;c__Verrucomicrobiae;o__Verrucomicrobiales;f__Verrucomicrobiaceae;g__Persicirhabdus
No.178	k__Bacteria;p__[Thermi];c__Deinococci;o__Deinococcales;f__Deinococcaceae;g__Deinococcus

Table S3. List of annotated metabolites and unknown signals from NMR measurements.

Annotated metabolites detected by 1D and 2D NMR measurements (Figures S5, S6) (a). List of alternative names given to unknown ^{1}H chemical shifts from urinary analysis data (b) and from fecal analysis data (c).

a

Metabolites		^{1}H Chemical Shift (ppm) and multiplicity
Acetic acid	Ac	1.91 (s)
Acetylglycine	AcGly	2.02 (s), 3.74 (d)
Adenosine monophosphate	3'-AMP	3.89 (m), 4.43 (m), 6.11 (d), 8.25 (s), 8.37 (s)
a-Hydroxyisobutyrate	HydroxyisoBuA	1.34 (s)
Allantoin	Allantoin	5.37 (s)
Betaine	Betaine	3.25 (s), 3.89 (s)
Butanoic acid	BuA	0.88 (t), 1.55 (q), 2.15 (t)
Cholic acid	Cholate	0.71 (s), 2.22 (m)
Choline	Choline	3.20 (s), 3.50 (dd), 4.05 (m)
Cis-Aconitic acid	Aconi	3.11 (d), 6.58 (s)
Citric acid	CiA	2.54 (d), 2.68 (d)
Creatine	Creatine	3.02 (s), 3.92 (s)
Creatinine	Creatinine	3.03 (s), 4.05 (s)
Cytidine Monophosphate	3'-CMP	3.98 (dd), 4.04 (dd), 4.23 (d), 4.36 (m), 5.93 (d), 6.13 (d), 8.08 (d)
Deoxyadenosine	Deoxyadenosine	2.56 (d), 2.82 (dd), 3.79 (dd), 3.84 (dd), 4.18 (q), 4.65 (m), 8.18 (s), 8.29 (s)
D-Glucose	Glc	3.24 (dd), 3.39 (m), 3.46 (m), 3.53 (dd), 3.70 (m), 3.83 (m), 3.89 (dd), 4.64 (d), 5.23 (d)
Dimethylamine	DMA	2.71 (s)
Dimethylglycine	DMG	2.88 (s)
Ethanol	EtOH	1.17 (t), 3.65 (q)
Ethanolamine	EtNH	3.13 (d), 3.81 (d)
Formic acid	FoA	8.45 (s)
Fumaric acid	FuA	6.51 (s)
Galactaric acid	Galactarate	3.93 (s), 4.26 (s)
Gamma-Aminobutyric acid	GABA	1.89 (m), 2.29 (t), 3.00 (t)
Gluconic acid	Gluconate	3.66 (m).3.76 (m), 3.82 (m), 4.12 (d)
Glucuronate	Glucuronate	3.28 (t), 3.57 (dd), 3.72 (m), 4.07 (d), 4.63 (d), 5.23 (d)
Glycine	Gly	3.55 (s)
Guanidinoacetic acid	GuaniAc	3.78 (s)
Guanosine	Guanosine	3.82 (d), 3.87 (d), 4.22 (q), 4.41 (dd), 5.09 (d)
Hippuric acid	Hippurate	3.95 (d), 7.54 (m), 7.62 (t), 7.82 (dd)
Homovanillic acid	Homovanillate	3.44 (s), 3.86 (s), 6.77 (dd), 6.87 (d), 6.96 (d)
Hypotaurine	Hypotaurine	2.64 (t), 3.34 (t)
Inosine	Inosine	3.83 (dd), 3.90 (dd), 4.26 (dd), 6.06 (d), 8.19 (s)
Inosinemonophosphate	IMP	4.03 (m), 4.36 (dd), 4.50 (dd), 6.14 (d), 8.21 (s)
Isovalerylglycine	IsovalerylGly	0.93 (d), 2.16 (d), 3.75 (d)
Itaconic acid	Itaconate	3.40 (s), 5.85 (s)
L-Acetylcarnithine	AcCarni	2.12 (s), 2.48 (dd), 2.61 (dd), 3.18 (s), 3.61 (d), 3.82 (dd)
Lactic acid	Lactate	1.32 (d), 4.10 (q)
L-Alanine	Ala	1.47 (d), 3.76 (q)
L-Asparagine	Asn	2.85 (m), 2.94 (m), 4.00 (dd)
L-Aspartic acid	Asp	2.67 (dd), 2.80 (dd), 3.89 (dd)
L-Glutamic acid	Glu	2.05 (m), 2.11 (m), 2.34 (m), 3.74 (dd)
L-Glutamine	Gln	2.12 (m), 2.44 (m), 3.76 (t)
L-Isoleucine	Ile	0.93 (t), 1.00 (d), 1.25 (m), 1.45 (m), 1.96 (m), 3.66 (d)
L-Leucine	Leu	0.95 (t), 1.70 (m), 3.73 (m)
L-Lysine	Lys	1.46 (m), 1.72 (m), 1.89 (m), 3.01 (t), 3.74 (t)
L-Methionine	Met	2.15 (m), 2.63 (t), 3.85 (dd)
L-Phenylalanine	Phe	3.19 (m), 3.98 (dd), 7.32 (d), 7.36 (m), 7.42 (m)
L-Serine	Ser	3.83 (dd), 3.96 (s)
L-Threonine	Thr	1.31 (d), 3.58 (d), 4.25 (m)
L-Tyrosine	Tyr	3.02 (dd), 3.17 (dd), 3.92 (dd), 6.89 (d), 7.17 (m)
L-Valine	Val	0.98 (d), 1.03 (d), 2.27 (m), 3.60 (d)
Methanol	MtOH	3.34 (s)
Methylamine	Methylamine	2.59 (s)
N-Acethyl	N-Act	2.05 (s)
Propionic acid	PrA	1.05 (t), 2.17 (q)
Putrescine	Putrescine	1.76 (t), 3.04 (t)
Sarcosine	Sarcosine	2.74 (s), 3.60 (s)
Succinic acid	SuA	2.40 (s)
Taurine	Taurine	3.26 (t), 3.42 (t)
Trigonelline	Trigonelline	4.43 (s), 8.07 (m), 8.82 (m), 9.11 (s)
Trimethylamine	TMA	2.88 (s)
Uridine	Uridine	3.80 (dd), 3.90 (dd), 4.12 (m), 4.22 (dd), 4.34 (dd), 5.89 (d), 5.90 (d), 7.86 (d)
Urocanic acid	Urocanate	6.39 (d), 7.28 (d), 7.37 (s), 7.85 (s)
Xylobiose	Xyl	2.91 (m), 3.22 (dd), 3.25 (m), 3.30 (m), 3.37 (dd), 3.42 (t), 3.54 (t), 3.62 (m), 3.68 (dd), 3.77 (m), 3.92 (dd), 3.97 (dd), 4.05 (dd), 4.45 (d), 4.58 (d)
2-oxoglutarate	2Oxoglutarate	2.42 (t), 3.00 (t)
2-oxoisovaleric acid	2Oxoisovalerate	0.82 (s), 0.95 (d), 2.01 (m), 3.84 (d)
2-oxopentanoic acid	2Oxopentanoate	0.91 (t), 1.61 (m)
3-ureidopropionate	3-UrPrA	2.72 (t), 3.29 (t)
4-amino-hippurate	4aminoHippurate	3.89 (d), 6.85 (d), 7.66 (d)

b

Unknown ^{1}H Chemical Shift (ppm)			
ROI.1	1.24	ROI.32	3.64
ROI.2	3.14	ROI.33	3.85
ROI.3	0.87	ROI.34	3.94
ROI.4	0.88	ROI.35	4.12
ROI.5	0.90	ROI.36	4.14
ROI.6	1.09	ROI.37	4.29
ROI.7	1.11	ROI.38	4.47
ROI.8	1.20	ROI.39	4.51
ROI.9	1.22	ROI.40	4.55
ROI.10	1.35	ROI.41	4.57
ROI.11	1.36	ROI.42	5.01
ROI.12	1.48	ROI.43	5.37
ROI.13	1.76	ROI.44	5.70
ROI.14	1.97	ROI.45	6.53
ROI.15	1.99	ROI.46	6.72
ROI.16	2.06	ROI.47	6.84
ROI.17	2.08	ROI.48	6.86
ROI.18	2.24	ROI.49	6.87
ROI.19	2.27	ROI.50	6.93
ROI.20	2.29	ROI.51	7.05
ROI.21	2.46	ROI.52	7.19
ROI.22	2.50	ROI.53	7.21
ROI.23	2.58	ROI.54	7.25
ROI.24	2.60	ROI.55	7.27
ROI.25	2.62	ROI.56	7.35
ROI.26	2.92	ROI.57	7.37
ROI.27	3.03	ROI.58	7.56
ROI.28	3.15	ROI.59	7.67
ROI.29	3.22	ROI.60	3.80
ROI.30	3.29	ROI.61	3.67
ROI.31	3.59	ROI.62	3.61

c

Unknown ^{1}H Chemical Shift (ppm)			
ROI.1	2.90	ROI.35	2.92
ROI.2	3.48	ROI.36	3.03
ROI.3	2.46	ROI.37	3.40
ROI.4	2.36	ROI.38	3.65
ROI.5	2.33	ROI.39	3.67
ROI.6	4.04	ROI.40	3.68
ROI.7	7.23	ROI.41	3.75
ROI.8	4.27	ROI.42	3.86
ROI.9	2.88	ROI.43	3.87
ROI.10	3.10	ROI.44	3.90
ROI.11	3.13	ROI.45	4.06
ROI.12	3.99	ROI.46	4.10
ROI.13	3.99	ROI.47	4.46
ROI.14	3.94	ROI.48	4.47
ROI.15	3.98	ROI.49	4.59
ROI.16	1.52	ROI.50	4.60
ROI.17	2.48	ROI.51	4.61
ROI.18	2.49	ROI.52	4.61
ROI.19	2.55	ROI.53	5.09
ROI.20	2.86	ROI.54	5.10
ROI.21	2.89	ROI.55	5.13
ROI.22	2.93	ROI.56	5.86
ROI.23	7.89	ROI.57	5.92
ROI.24	7.85	ROI.58	6.05
ROI.25	5.18	ROI.59	6.06
ROI.26	1.13	ROI.60	6.07
ROI.27	1.16	ROI.61	6.13
ROI.28	1.19	ROI.62	6.82
ROI.29	1.20	ROI.63	7.73
ROI.30	1.36	ROI.64	7.67
ROI.31	1.37	ROI.65	7.91
ROI.32	2.61	ROI.66	7.96
ROI.33	2.71	ROI.67	8.59
ROI.34	2.73		

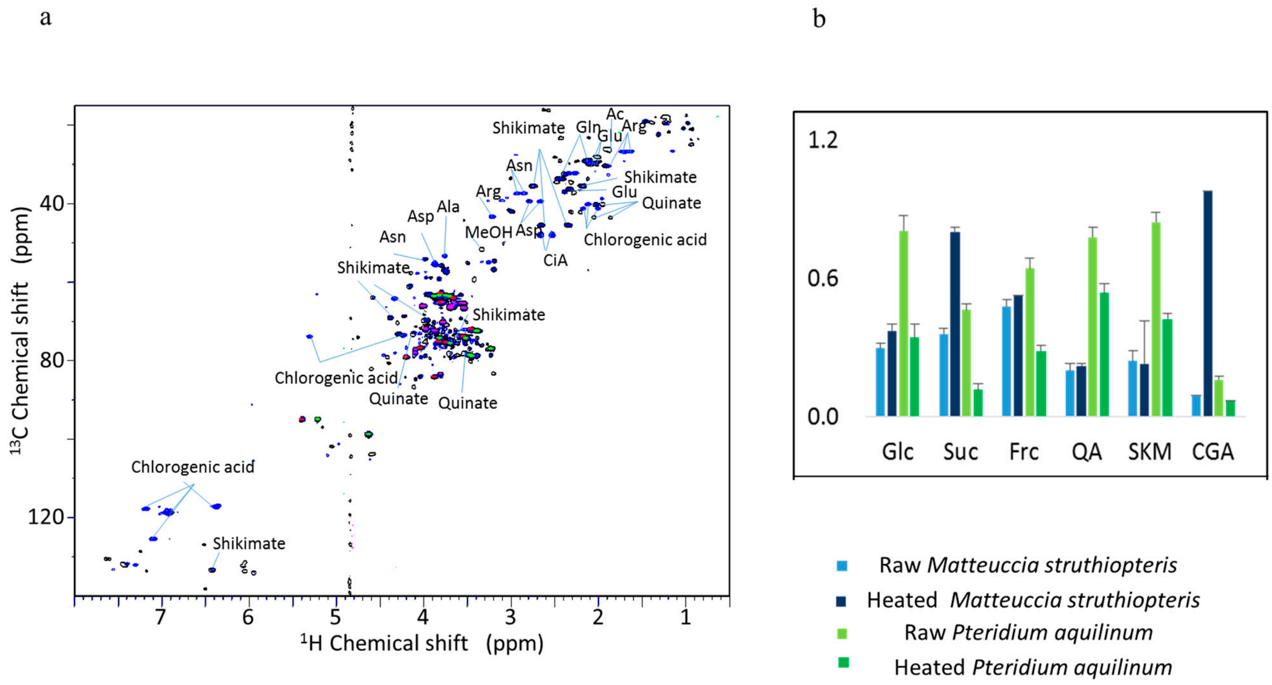

Figure S3. ^{1}H-^{13}C HSQC NMR spectra of *Pteridium aquilinum* and *Matteuccia struthiopteris* extracted by KPi/D_2O solvent. (a) Black and blue line spectra are from *P. aquilinum* and *M. struthiopteris*, respectively. Green, red, and purple spectra are derived from glucose, sucrose, and fructose, respectively. Annotations for the referenced metabolites listed in Table S3. (b) Sugar group and organic acid compositions in raw or boiled *P. aquilinum* and *M. struthiopteris* are shown as a bar graph.

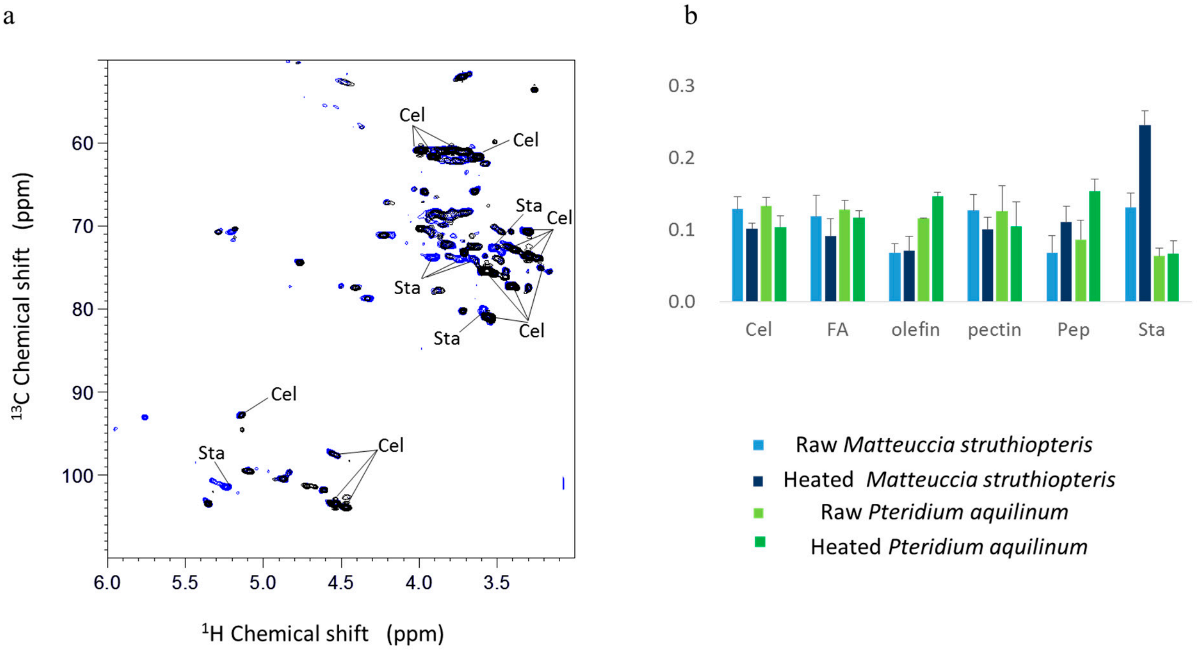

Figure S4. ^{1}H-^{13}C HSQC NMR spectra of *Pteridium aquilinum* and *Matteuccia struthiopteris* components extracted by DMSO-pyridine-d_5 (4:1) solvent. (a) Black and blue line spectra represent *Pteridium aquilinum* and *Matteuccia struthiopteris*, respectively. (b) Carbohydrate compositions in raw or boiled *P. aquilinum* and *M. struthiopteris* are shown as a bar graph.

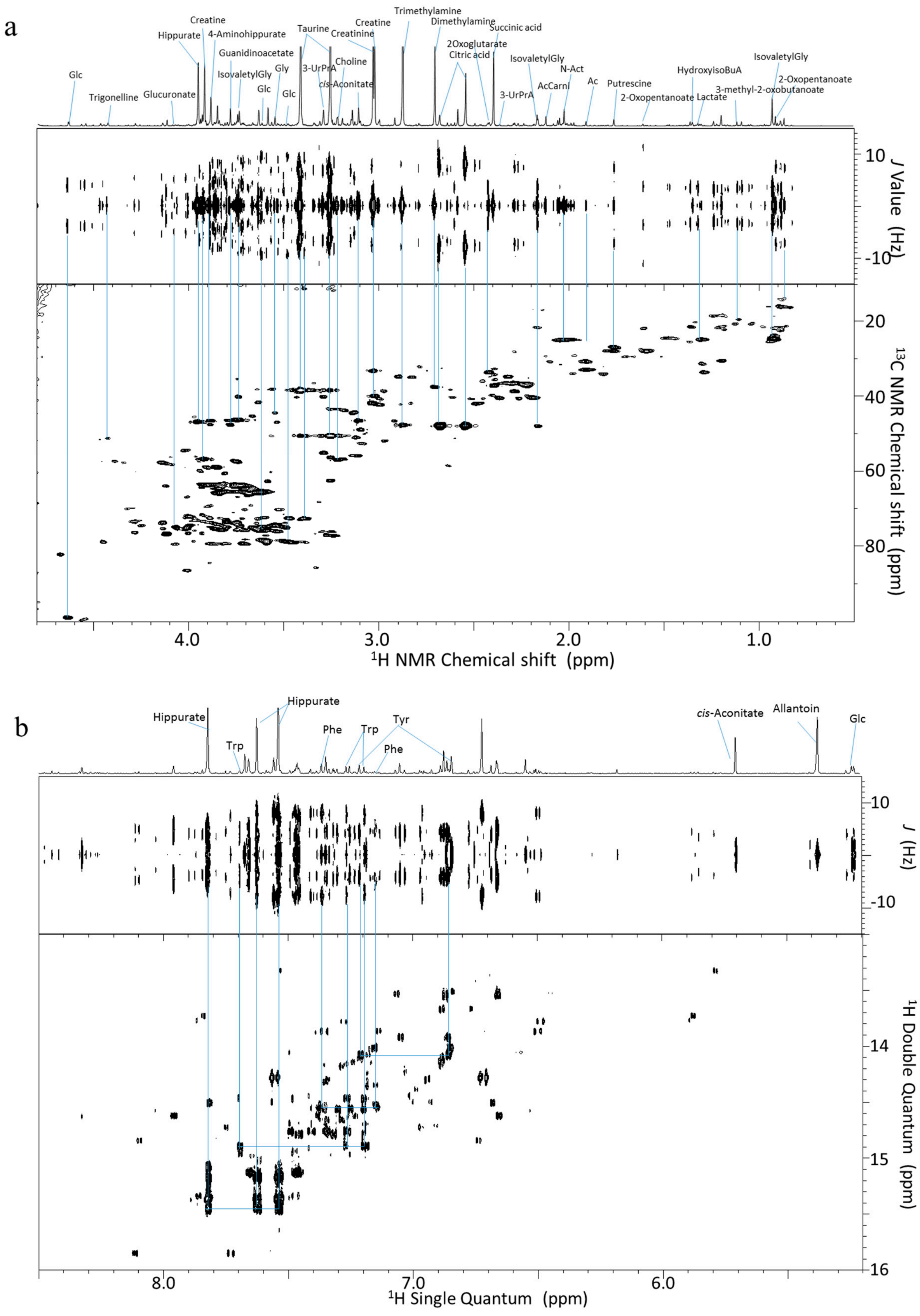

Figure S5. Annotation of metabolites in urine NMR spectra. Expanded version of ^{1}H NMR spectra from treated mouse urine (top panel), 2D *J*-resolved NMR spectra (middle panel), and DQ-SQ NMR spectra (bottom panel) from 0.5 to 4.8 ppm (a), and 5.2 to 8.5 ppm (b). Annotations for the referenced metabolites listed in Table S3.

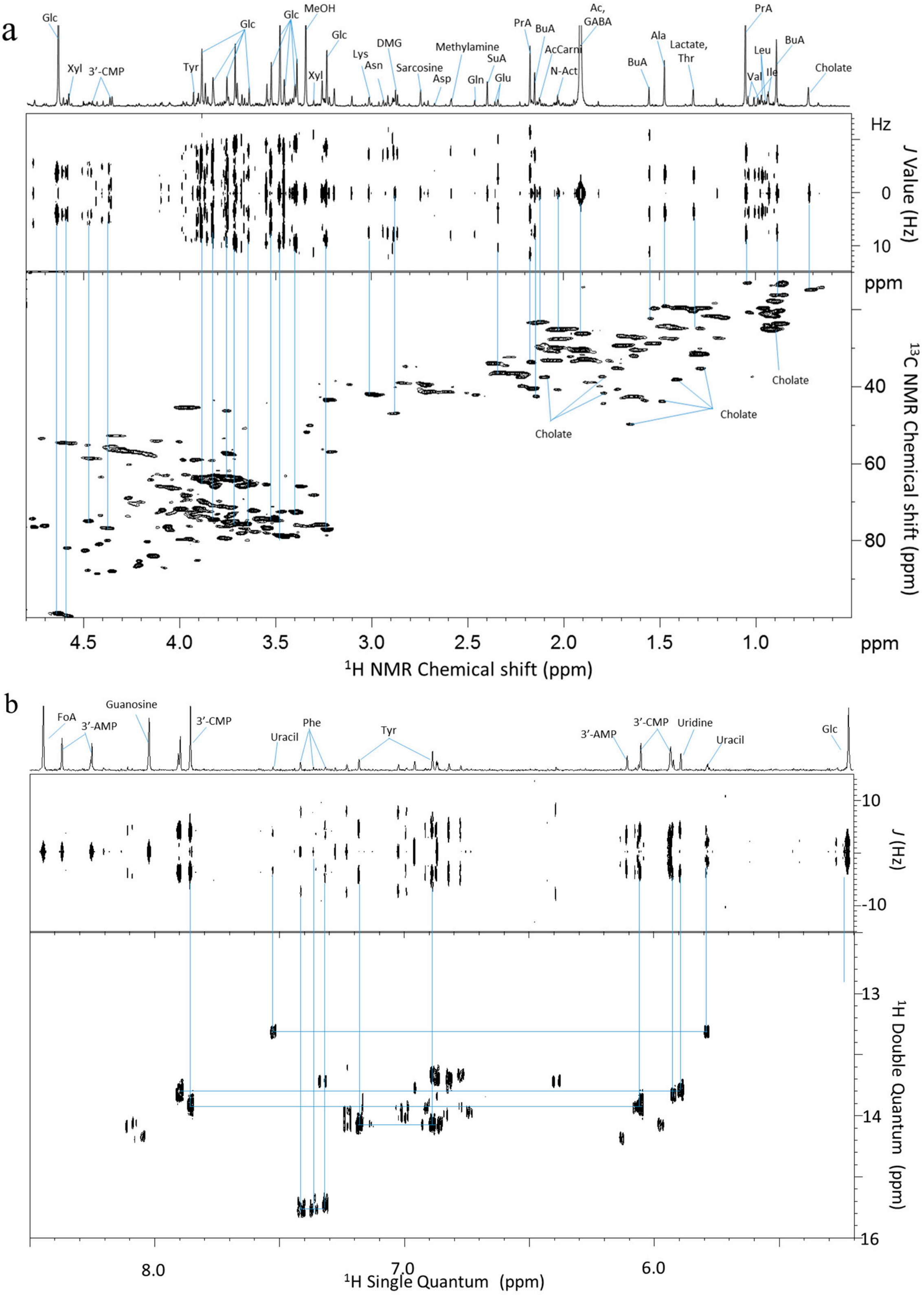

Figure S6. Annotation of metabolites in fecal NMR spectra. Expanded ^{1}H NMR spectra from treated mouse feces (top panel), 2D *J*-resolved NMR spectra (middle panel), and DQ-SQ NMR spectra (bottom panel) from 0.5 to 4.8 ppm (a), and 5.2 to 8.5 ppm (b). Annotations for referenced metabolites listed in Table S3.

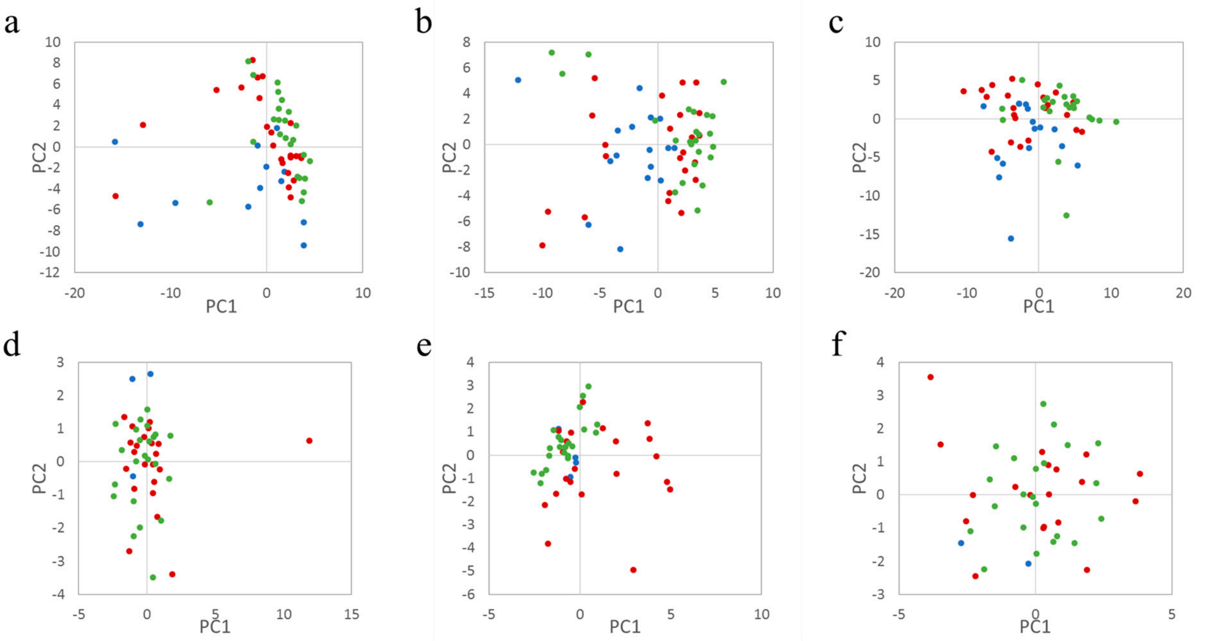

Figure S7. Principal component analysis of urinary metabolites and ions. Red, blue, and green spheres represent single data points from pretreatment, during treatment, and posttreatment, respectively. Upper panels indicate the results of urinary NMR measurement analyzed by PCA. The data based on experimentally treated mouse groups (a–c) administered cellulose, *Pteridium aquilinum*, and *Matteuccia struthiopteris*, respectively. (d–f) show PCA score plots for the ICP-OES data from each group. (d–f) represent ions of mice treated with cellulose, *Pteridium aquilinum*, and *M. struthiopteris* measured by ICP-OES, respectively. The data analysis was performed using the package "muma" in R [45].

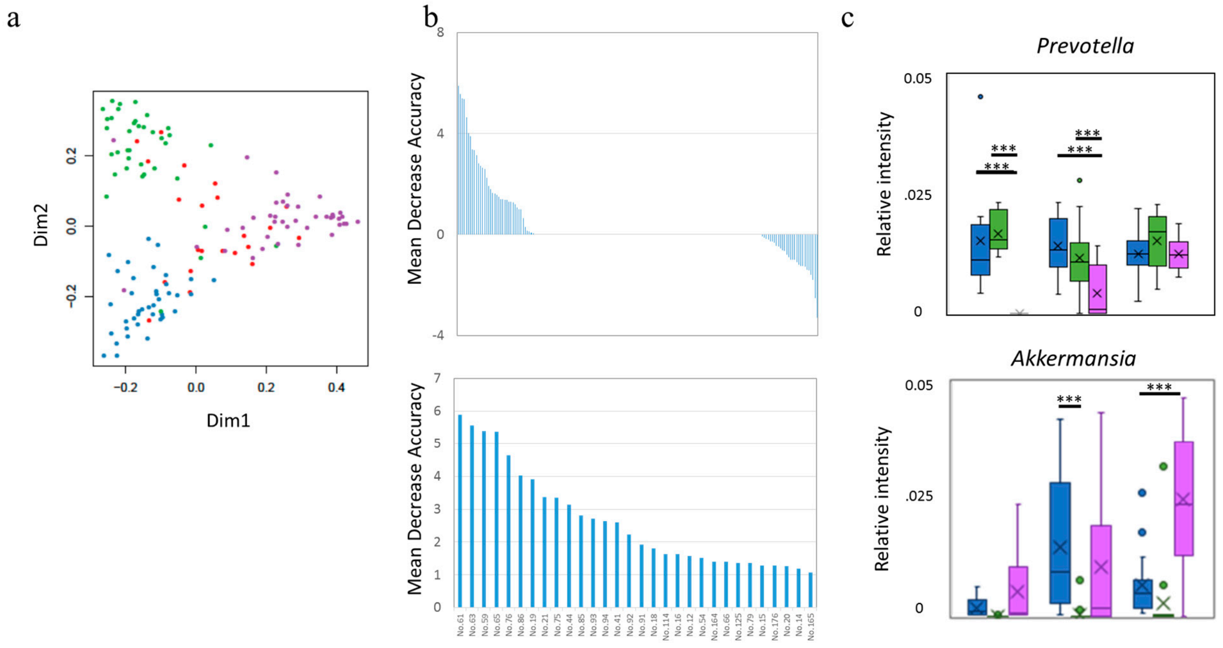

Figure S8. The results of Random Forest for the fecal microbiome. MDS plot (a) and factors important for separating the groups (b): upper panel indicates all important factors, while lower panel indicates the top 30 most important factors with annotation. In (a), red, blue, green, and purple spheres represent single data points from the pretreatment period of all groups, and cellulose-, *Pteridium aquilinum*-, and *Matteuccia struthiopteris*-treated groups, respectively. Boxplot of a representative important factor that can be applied on to network module (c). Upper panel and lower panel depict the genera *Prevotella* and *Akkermansia*, respectively. Blue, green, and purple boxes represent cellulose-, *P. aquilinum*-, and *M. struthiopteris*-treated groups, respectively. *** P < 0.001 calculated by the Holm method.

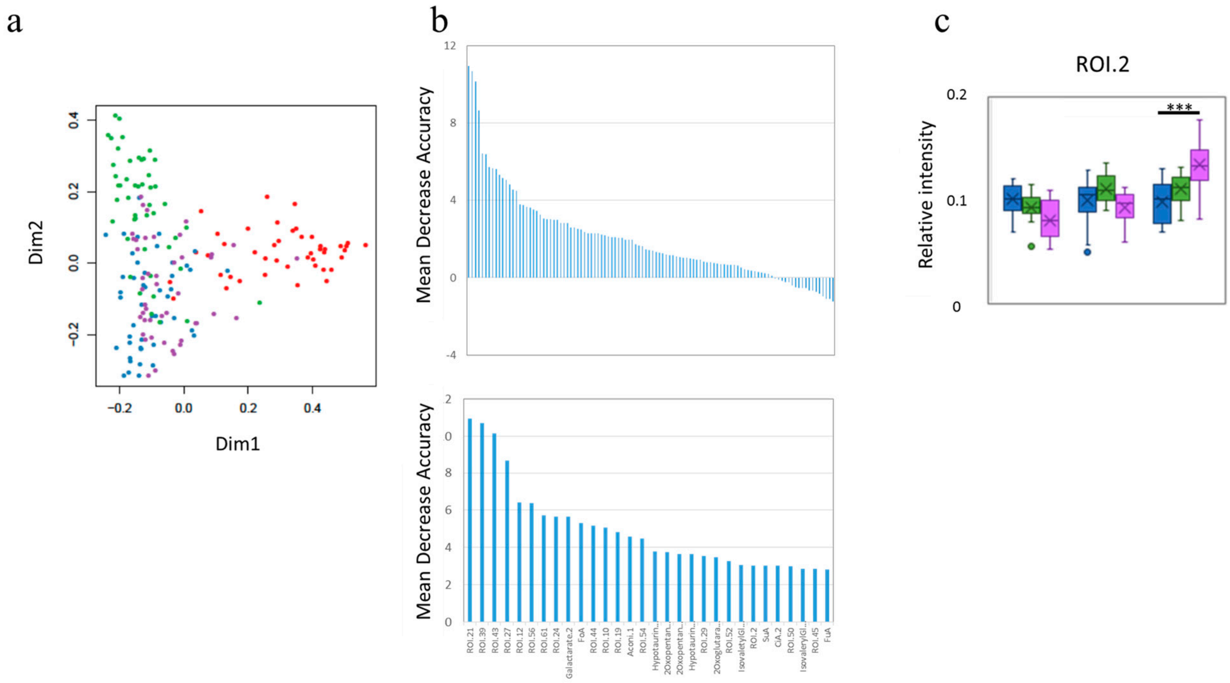

Figure S9. The results of Random Forest for urinary metabolites. MDS plot (a) and factors important for separating the groups (b): upper panel indicates all important factors, while lower panel indicates the top 30 most important factors with annotation. In (a), red, blue, green, and purple spheres represent single data points from the pretreatment period of all groups, and cellulose-, *Pteridium aquilinum*-, and *Matteuccia struthiopteris*-treated groups, respectively. Boxplot of a representative important factor that can be applied onto network module, which depicts ROI.2 (c). Blue, green, and purple boxes represent cellulose-, *P. aquilinum*-, and *M. struthiopteris*-treated groups, respectively. *** P < 0.001 calculated by the Holm method.

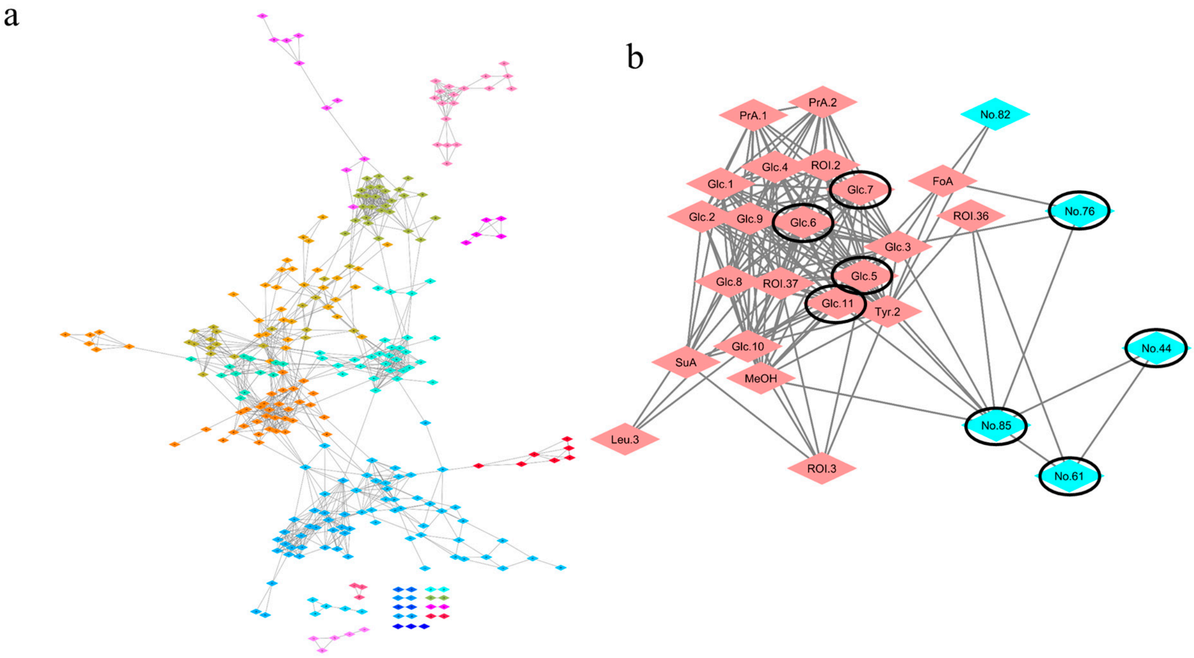

Figure S10. Network community correlation coefficients for *Matteuccia struthiopteris*-treated mice based on the combined data set. The network depiction is based on calculations by the igraph package with Cytoscape. All communities of *Matteuccia struthiopteris*-treated mice are depicted (a). The same color diamonds represent components belonging to the same community. The glucose community in *M. struthiopteris*-treated mice is depicted (b). Black-line-circled diamonds represent factors selected by Random Forest as being important for separating the groups (Table S3).

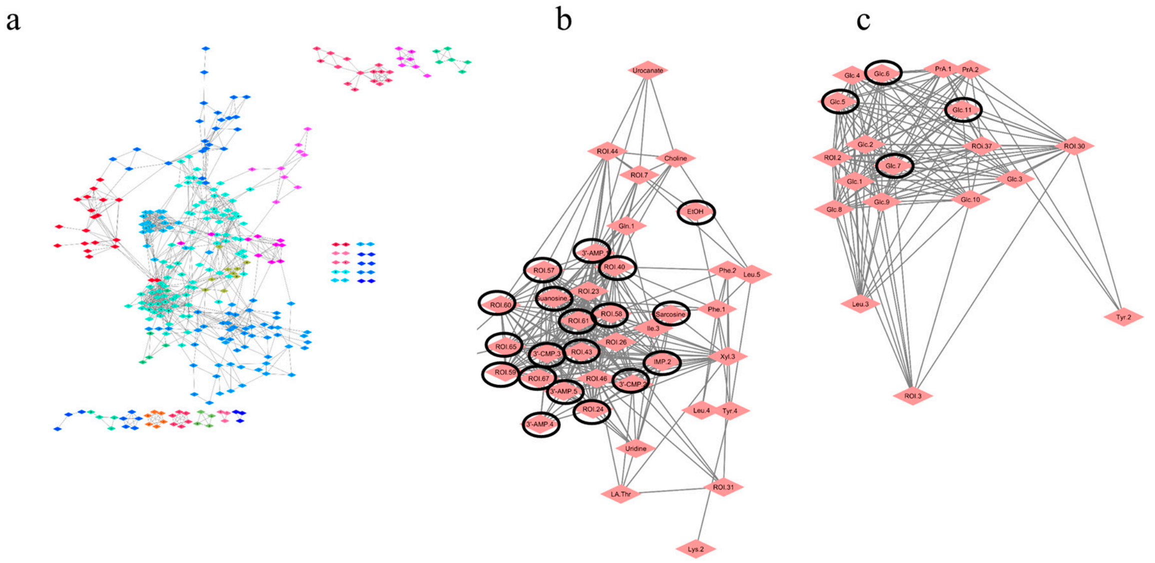

Figure S11. Network community correlation coefficients for *Pteridium aquilinum*-treated mice based on the combined data set. The network depiction is based on calculations by the igraph package with Cytoscape. All communities of *P. aquilinum*-treated mice are depicted (a). The same color diamonds represent components belonging to the same community. Purine/pyrimidine and glucose communities in *Matteuccia struthiopteris*-treated mice are indicated in (b) and (c), respectively. Black-line-circled diamonds represent factors selected by Random Forest as being important for separating the groups (TableS3).
